# Treatment with β-Adrenoceptor Agonist Isoproterenol Reduces Non-parenchymal Cell Responses in LPS/D-GalN-Induced Liver Injury

**DOI:** 10.1007/s10753-023-01941-z

**Published:** 2023-12-21

**Authors:** Yuchao Wu, Tianzhi Ni, Mengmeng Zhang, Shan Fu, Danfeng Ren, Yali Feng, Huiping Liang, Ze Zhang, Yingren Zhao, Yingli He, Yuan Yang, Zhen Tian, Taotao Yan, Jinfeng Liu

**Affiliations:** 1 Department of Infectious Diseases and Hepatopathy, The First Affiliated Hospital of Xi’an Jiaotong University, Xi’an City, China; 2 Department of Radiation Oncology, Affiliated Cancer Hospital & Institute of Guangzhou Medical University, Guangzhou, Guangdong Province, China; 3 Honghui Hospital, Xi’an Jiaotong University, Xi’an City, China; 4 Department of Ultrasound, The First Affiliated Hospital of Xi’an Jiaotong University, Xi’an City, Shaanxi Province China

**Keywords:** proteomic, hepatic non-parenchymal cells, isoproterenol, acute liver failure.

## Abstract

There is an increasing evidence indicating the involvement of the sympathetic nervous system (SNS) in liver disease development. To achieve an extensive comprehension of the obscure process by which the SNS alleviates inflammatory damage in non-parenchymal liver cells (NPCs) during acute liver failure (ALF), we employ isoproterenol (ISO), a beta-adrenoceptor agonist, to mimic SNS signaling. ISO was administered to C57BL/6J mice to establish an acute liver failure (ALF) model using LPS/D-GalN, which was defined as ISO + ALF. Non-parenchymal cells (NPCs) were isolated from liver tissues and digested for tandem mass tag (TMT) labeled proteomics to identify differentially expressed proteins (DEPs). The administration of ISO resulted in a decreased serum levels of pro-inflammatory cytokines, *e.g.*, TNF-α, IL-1β, and IL-6 in ALF mice, which alleviated liver damage. By using TMT analysis, it was possible to identify 1587 differentially expressed proteins (DEPs) in isolated NPCs. Notably, over 60% of the DEPs in the ISO + ALF *vs.* ALF comparison were shared in the Con *vs.* ALF comparison. According to enrichment analysis, the DEPs influenced by ISO in ALF mice were linked to biological functions of heme and fatty acid metabolism, interferon gamma response, TNFA signaling pathway, and mitochondrial oxidation function. Protein-protein interaction network analysis indicated Mapk14 and Caspase3 may serve as potentially valuable indicators of ISO intervention. In addition, the markers on activated macrophages, such as Mapk14, Casp1, Casp8, and Mrc1, were identified downregulated after ISO initiation. ISO treatment increased the abundance of anti-inflammatory markers in mouse macrophages, as evidenced by the immunohistochemistry (IHC) slides showing an increase in Arg + staining and a reduction in iNOS + staining. Furthermore, pretreatment with ISO also resulted in a reduction of LPS-stimulated inflammation signaling markers, Mapk14 and NF-κB, in human THP-1 cells. Prior treatment with ISO may have the potential to modify the biological functions of NPCs and could serve as an innovative pharmacotherapy for delaying the pathogenesis and progression of ALF.

**Supplementary Information:**

The online version contains supplementary material available at 10.1007/s10753-023-01941-z.

## Introduction

Acute liver failure (ALF) is a potentially deadly ailment characterized by unmanageable inflammation within the liver, rapid deterioration of liver function, and extensive hepatocyte destruction. Due to the severity and diversity of the clinical syndrome, it is challenging to discover effective treatments. This circumstance increases mortality rates in intensive care units in the short term [1, 2].

The liver is a multifaceted organ comprised of diverse cell types that collaborate to execute critical metabolic and immunological functions. Previous studies have shown that non-parenchymal cells in the liver, including Kupffer cells, hepatic stellate cells, liver sinusoidal endothelial cells, and other immune cells, have a significant impact on the pathogenesis of acute liver failure. This is either through direct interaction with parenchymal hepatocytes in hepatic sinusoids or by producing various inflammatory mediators, cytokines, oxidative stress products, microRNAs, and lipid metabolites. A comprehensive understanding of the functions performed by hepatic non-parenchymal cells in acute liver failure (ALF) is crucial for the advancement of innovative treatment methods [3, 4].

As the autonomic nervous system, consisting of both the sympathetic and parasympathetic branches, plays an essential role in maintaining liver tissue homeostasis, researchers have been focusing on it recently [5–7]. It is interesting to note that the hepatic sympathetic nervous system (SNS) controls several liver ailments, such as non-alcoholic fatty liver disease and cirrhosis [7–9]. Agents targeting the alpha- and beta-adrenergic receptors, which are vital for neurotransmission and regulate the sympathetic nervous system, demonstrate effects on liver injury models. For example, prazosin effectively reduces liver injury by blocking alpha-adrenergic receptors, while propranolol, a beta-adrenergic receptor antagonist, can exacerbate it [10]. Specifically, substances that stimulate the adrenergic system and its receptors have the potential to encourage growth of precursor cells and hepatocytes and also provide defense against sudden liver damage [10–12]. However, the proliferation of progenitor cells accounts for only a small proportion of liver cells, while the vast majority of non-parenchymal hepatic cells, including Kupffer cells, are also important in modulating the progression of inflammation. The precise impact of adrenergic receptors on hepatic non-parenchymal cells during the inflammatory phase of ALF remains uncertain. To achieve this, we investigated the biochemical impacts of β-adrenoceptors on non-parenchymal liver cells by administering the β-adrenoceptor agonist isoproterenol (ISO) in an animal model of ALF.

## Materials and Methods

### Animal Studies

The C57BL/6 J female/male mice weighed 20 ± 2 g and aged 6–8 weeks old were obtained from Xi’an Jiaotong University’s Animal Experimentation Centre. All mice were housed in a pathogen-free environment with a temperature range of 23 ± 2 °C, humidity between 50 and 70%, light and dark cycles lasting 12 h, and unlimited access to food and water. The animal care and experimental procedures underwent approval from the Xi’an Jiaotong University Animal Experiment Ethics Committee and Authority, as evidenced by Approval No. 2022–390.

To establish the ISO + ALF group, ISO (10 mg/kg) was administered intraperitoneally 1 h prior to LPS/D-GalN injection, corresponding to published dosages for liver disease [10, 13]. In addition, we conducted experiment with different doses of mere isoproterenol, 0, 10, and 100 mg/kg, injected into mouse. These experiments preliminarily suggested that ISO had no significant impact on liver structure or hepatotoxicity (Fig. S1).

According to established procedures [14], the ALF model was induced in C57BL/6 J mice by intraperitoneal administration of LPS (30 mg/kg, Sigma, USA) and D-galactosamine (600 mg/kg, Sigma, USA). The mice were monitored in a controlled environment for a maximum of 12 h. Afterward, all the mice were humanely euthanized, and their liver tissue and serum samples were collected for further analysis of the results.

### Cell Culture and Treatment

THP-1, a cell line of human macrophages, was acquired from the National Collection of Authenticated Cell Cultures of China. The cells were grown in 1640 medium with 10% fetal bovine serum and 1% penicillin/streptomycin added. The incubator was kept in an environment with 5% CO_2_ at 37 °C. THP-1 cells were exposed to LPS (20 ng/ml; Sigma) for 2 h in order to cause inflammation. Two hours before LPS stimulation, ISO (at a dose of 50 µM; MCE) was given after being dissolved in DMSO. Following the extraction of cell lysis and collection of mRNA, the samples were collected and kept at −80 °C until they were needed.

### NPCs Isolation

According to previously published protocols [15, 16], hepatic non-parenchymal cells (NPCs) were obtained from liver tissues by collagenase type IV digestion (0.05 mg/mL) and gradient centrifugation. In brief, liver tissues were infused with perfusion buffer for up to 20 min then perfused with collagenase until digested. Collect the cell suspension and filter into 50-ml plastic tubes through a gauze-lined plastic funnel. Add more stop solution to the sample of liver tissue until a final volume of 500 ml is reached. Centrifuge the cell suspension at 50× g for 5 min at 4 °C for subsequent isolation of non-parenchymal cells, collect the supernatant. Wash the cell pellet with PBS and collect for hepatocyte. Centrifuge the harvested supernatant at 72× g for 5 min at 4 °C to remove any remaining red blood cells and liver cells. Pool the supernatants and centrifuge them twice. Combine both pellets and resuspend them in HBSS. Prepare a 25% and a 50% density gradient by mixing the density gradient solution with PBS for density gradient centrifugation. Carefully place the 25% density gradient solution on top of the layer containing the 50% density gradient solution. Then, add the NPC suspension slowly on top of the 25% density gradient solution to ensure a clear separation between the layers is achieved. Centrifuge the cell suspension on the density gradient at 1800× g for 20 min at 4 °C without applying any brake. Remove dead cells and cell debris from the topmost layer. The non-parenchymal cells can be found in the interphase between the 25 and 50% density gradient layers. Collect the cell suspension using the dual centrifugation step described above and wash them with Hank’s balanced salt solution (HBSS) and centrifuge. Store the collected NPCs at −80 °C until further use.

### Protein Extraction and Digestion for Proteomics Analysis

After isolating the sample, liquid nitrogen was served to grind the cells into powder, and the powder was then transferred to a 5-mL centrifuge tube. Following this, four volumes of lysis buffer (8 M urea, 1% protease inhibitor cocktail) were added to the cell powder and subjected to three rounds of ultrasonication on ice using a high intensity ultrasonic processor (Scientz). In the case of PTM experiments, inhibitors were additionally included in the lysis buffer, for example. For acetylation, a concentration of 3 µM TSA and 50 mM NAM was utilized, while for phosphorylation, 1% phosphatase inhibitor was added. Centrifugation at 12,000 g at 4 °C for 10 min effectively eliminated the remaining debris. Afterward, the liquid above the sediment was gathered and the amount of protein was measured using the BCA kit as per the instructions supplied by the manufacturer.

For the process of digestion, the protein solution underwent reduction using a 5 mM dithiothreitol solution for a duration of 30 min at a temperature of 56 °C. Subsequently, the solution was alkylated with an 11 mM iodoacetamide solution for a duration of 15 min at room temperature in a dark environment. To decrease the concentration of urea in the protein sample to less than 2 M, it was diluted by the addition of a 100 mM TEAB solution. Following this, trypsin was introduced at a trypsin-to-protein mass ratio of 1:50 for the initial overnight digestion and at a ratio of 1:100 for a subsequent 4-h digestion. Finally, the peptides were purified through the usage of a C18 solid-phase extraction column.

### TMT Labeling and HPLC Fractionation

During the TMT labeling procedure, the tryptic peptides were first dissolved in a 0.5 M TEAB solution. Then, each individual peptide channel was labeled using the appropriate TMT reagent (Thermo Fisher Scientific) in accordance with the manufacturer’s instructions, and it was incubated for 2 h at room temperature. Five microliters of each sample was mixed, purified, and then exposed to MS analysis in order to evaluate the labeling efficiency. After the efficacy of the labeling was evaluated, 5% hydroxylamine was added to the samples to quench them. The mixed samples were then dried by vacuum centrifugation after being desalted using a Phenomenex Strata X C18 SPE column.

The sample was subjected to HPLC grading, wherein it was fractionated into distinct fractions using a high pH reverse-phase HPLC method employing an Agilent 300 Extend C18 column (consisting of 5 µm particles, with an inner diameter of 4.6 mm and a length of 250 mm). In summary, peptides were separated by employing a gradient of acetonitrile ranging from 2 to 60% in a 10 mM ammonium bicarbonate solution at pH 10, over a duration of 80 min, resulting in the generation of 80 fractions. Subsequently, the peptides were consolidated into 9 fractions and subsequently dried through vacuum centrifugation.

### LC–MS/MS Analysis

The tryptic peptides were dissolved in solvent A, which consisted of 0.1% formic acid and 2% acetonitrile in water. Subsequently, they were loaded on a reversed-phase analytical column that was 25 cm in length and had an inner diameter of 75 µm. The peptides were isolated using a gradual change in solvent B (0.1% formic acid in 90% acetonitrile) over a period of 60 min, followed by a transition from 25 to 35% in 22 min and a rapid increase to 80% in 4 min. The concentration was maintained at 80% for the final 4 min, while the flowrate remained constant at 450 nL/min on an EASY-nLC 1200 UPLC system (Thermo Fisher Scientific). The peptides that had been separated were examined using a nano-electrospray ion source in QExactiveTM HF-X (Thermo Fisher Scientific). A voltage of 2.0 kV was applied for electrospray. For a scan range of 350–1600 m/z, the complete MS scan resolution was adjusted to 60,000. 30 s dynamic exclusion was used to select the top 20 most prevalent precursors for subsequent MS/MS analyses. The fragmentation of HCD was carried out with a collision energy (NCE) of 28%. Fragments were identified in the Orbitrap with a resolution of 30,000. The initial mass was fixed at 100 m/z. The AGC target for automatic control was established at 1E5, with a threshold of 3.3E4 for intensity and a maximum injection time of 60 ms.

### Identification and Quantitation of Proteins

MaxQuant search engine (v.1.6.15.0) was utilized to process the above LC–MS/MS data. The human SwissProt database (20422 entries) was used to match tandem mass spectra. In the initial search, the tolerance for precursor ions was set at 20 ppm, while it reduced to 5 ppm in the primary search. Additionally, the tolerance for fragment ions was defined as 0.02 Da. Carbamidomethyl on Cys was referred to as a fixed modification, while acetylation on protein N-terminal and oxidation on Met were referred to as variable modifications. The adjustment of FDR was made to less than 0.001.

### Gene Set Enrichment Analysis

Using gene set enrichment analysis (GSEA) with the gene sets of mh.all.v2022.1 and Mm.symbols.gmt as references, the potential pathway from selected gene lists was examined. Protein sets input was classified as highly enriched if they had a *P* value < 0.05, a normalized enrichment score (NES) > 1 or < −1, and a False Discovery Rate (FDR) < 0.25. Data visualization was created using the clusterProfiler and ggplot2 packages in R (version 4.2.1).

### Bioinformatic Analysis

The protein list was analyzed by using DEqMS package in R to determine differentially expressed proteins (DEPs). DEPs were expressed with a *P* value < 0.05, fold change > 1.5 or < 0.75 and false discovery rate (FDR) < 0.01 within each group. GO (https://www.geneontology.org) and KEGG (https://www.genome.jp/kegg/) enrichment analysis was conducted to analyze the biological functions of DEPs through DAVID Functional Annotation Dataset (ncifcrf.gov) [17, 18]. GO enrichment analysis was performed for three ontologies, including biological process, molecular function, and cellular component. A significant GO term or pathway was identified with a threshold of protein number more than two and *P* value less than 0.05. Protein-protein interaction (PPI) analysis was constructed by using the String (https://string.embl.de/) database and Cytoscape (https://www.cytoscape.org/) software. Furthermore, we utilized ClusterViz (V. 1.0.3, Bochum, Germany) in Cytoscape to visualize the clustering analysis based on the molecular complex detection (MCODE) algorithm. We established the criteria as a degree cut-off of 2 and a node score cut-off as 0.2, for the MCODE algorithm, with a maximum depth as 100 k-score as 2 and high confidence as no less than 0.7. Additionally, rank abundance curves, clustering heatmaps, bubble plots, and Venn diagrams were carried out by using the R package in version 4.2.1. Specially, a hierarchical K-means cluster was used to analyze DEP heatmaps [19].

### RNA Extraction and qRT-PCR Analysis

RNA extraction and quantitative real-time PCR (qRT-PCR) were conducted following previously established protocols. Briefly, total RNA was extracted from frozen liver or cultured cells using TRIzol reagent (Thermo, Life Technologies, Carlsbad, CA, USA). The concentration and quality of the RNA were assessed, and mRNA was subsequently reverse transcribed into cDNA using the RevertAid First Strand cDNA Synthesis Kit (Thermo Scientific, Rockford, AL, USA). Subsequently, the relative levels of mRNA transcripts were determined through qRT-PCR analysis using the SYBR Premix ExTaq^™^II kit (TaKaRa, Dalian, China) on an iQTM multicolor real-time PCR detection system (Bio-Rad, Hercules, CA, USA). The 2^−ΔΔCT^ method was applied to calculate the relative expression of each gene using either actin or Glyceraldehyde-3-phosphate dehydrogenase as internal standards. Three technical replicates and three biological replicates were used for each PCR reaction.

### Western Blotting

RIPA lysis buffer supplemented with protease inhibitor cocktail tablets (Roche) and phosSTOP phosphatase inhibitor cocktails (Roche) was prepared to extracts total protein from cells or liver tissues. After being quantified using BCA assay, equal quantities of protein (30–50 µg) were loaded onto SDS/PAGE gels, separated, and then transferred onto PVDF membranes. After that, the membranes were blocked for 1 h at room temperature in a blocking buffer containing 5% non-fat milk. After an overnight incubation at 4 °C with certain primary antibodies, the blocked membranes were treated with secondary antibodies that were conjugated with HRP. Finally, the blots were visualized using ImageQuant LAS-4000 (Fujifilm) on a standard exposure setting and quantified using ImageJ (rsb.info.nih.gov/ij).

### Histological Examination

Samples of liver tissue taken from each group were immediately preserved in paraformaldehyde and then covered in paraffin. Using a microtome, tissues were divided into 4–5-µm sections and placed on glass microscope slides. After routine staining with hemotoxylin-eosin (H&E), two independent researchers analyzed the slides using light microscopy (DIALUX 20 EB).

### Flow Cytometry

The surface marker on THP-1 cells in both the resting (M0) and M1 polarized states was examined by using flow cytometry. The cells were lightly scraped after the 24-h stimulation of LPS or ISO, freshened three times with PBS, and then transferred into FACS tubes. The M0 and M1 phenotypes were then identified by adding surface CD40 (FITC, Biolegend) and CD14 (Pacific blue, Biolegend) fluorochrome-labeled monoclonal antibodies. Following a 4-h incubation period at 4 °C, the cells were subjected to three PBS washes before being analyzed using a BD Biosciences FACSCanto II flow cytometer. Each sample was recorded with at least 20,000 events. Each group was measured using the FlowJo program (Version 10.0), and at least three duplicates of each group were done.

### Statistical Analysis

Statistical data is presented as means ± standard deviation. Statistical differences among various treatment groups were conducted using one-way analysis of variance (ANOVA) with Tukey’s test in SPSS 13.0 (Chicago, IL, USA) or GraphPad Prism 5. Statistical significance was ascertained by considering two-tailed* P* value with a threshold of < 0.05.

## Results

### ISO Alleviated Pathological Liver Injury Induced by LPS/D-GalN

**Fig. 1:**
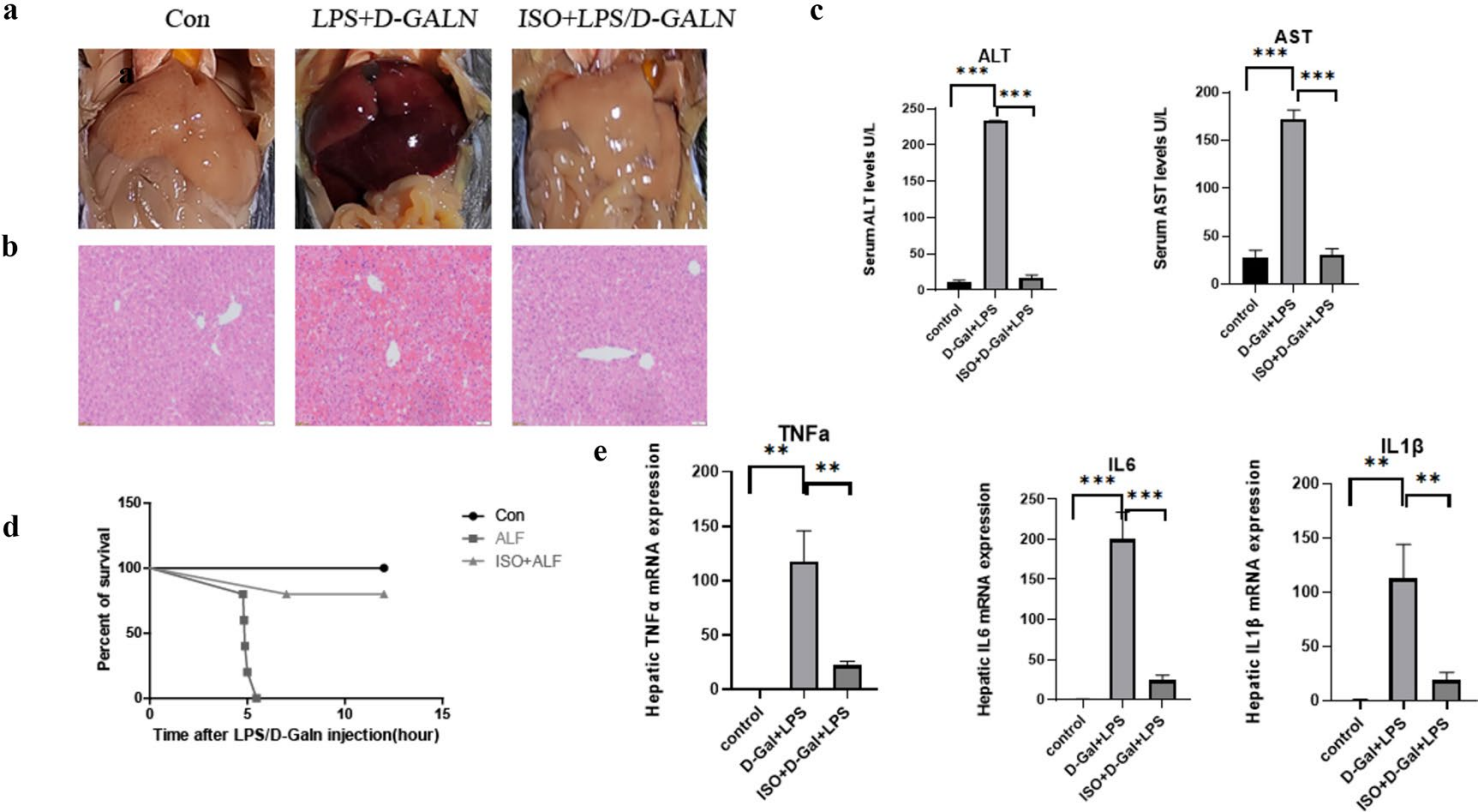
Effect of ISO treatment on liver hepatic pathology caused by lipopolysaccharide (LPS)/D-galactosamine (D-GalN) in female mice. **a** Morphological observation of liver tissue. **b** Representative sections of hematoxylin–eosin (H&E) staining (100-fold) in liver tissues. **c** Serum levels of alanine transaminase (ALT) and aspartate transaminase (AST), in mice with LPS/D-GalN administration. **d** Survival curves in different group of mice and **e** biological analysis of TNF-α, IL-6, and IL-1β mRNA levels in liver tissues. Statistical difference was performed by Student’s *t*-test in each comparison. **P < 0.01, ***P < 0.001.

Acute liver failure was induced in female mice by intraperitoneally co-administering LPS (30 mg/kg, Sigma, USA) and D-galactosamine (600 mg/kg, Sigma, USA), while equal does of PBS was given to control group. As revealed by gross morphology, the massive hepatic injury was apparent in 4~6 h. Hematoxylin–eosin (H&E) staining showed there are a lot of visible necrosis areas, inflammatory cell infiltration, and congestion in the liver tissue (Fig. 1a). Fascinatingly, we saw notable improvements in the pathology of the liver after pretreating the ALF mice with ISO (10 mg/kg) for 1 h. These improvements included a decrease in hepatocellular necrosis and a reduction in hyperemia and edema (Fig. 1b). Pre-treatment with ISO can reduce the increase in blood levels of alanine aminotransferase (ALT) and aspartate aminotransferase (AST) induced by LPS/D-GalN in ALF mice (Fig. 1c). These results imply that ISO may have a protective effect against liver injury and mortality caused by LPS/D-GalN. Following LPS/D-GalN injection, we compared survival rates of mice receiving ISO treatment with those receiving no treatment. As anticipated, the injection of LPS/D-GalN led to complete mortality within 12 h. However, prior administration of ISO resulted in lower death rates in ALF animals (refer to Fig. 1d). Additionally, we investigated the impact of ISO on the release of inflammatory cytokines induced by LPS/D-GalN. Subsequent to ISO therapy, there was a substantial decrease in the levels of TNF-α, IL-6, and IL1-β mRNAs (refer to Fig. 1e, *P* < 0.05). Overall, the results indicate that the LPS/D-GalN-based ALF mouse model employed in this study yielded favorable outcomes, and ISO displayed a beneficial impact in preventing liver damage induced by LPS/D-GalN.

The effects of sex-related differences on various diseases are intricate in humans and dependent on models in experimental laboratory animals. We sought to investigate the role of sex differences in the sympathetic system on regulating LPS-induced liver injury. We performed analogous experiments on male C57BL/6 J mice as we did on females. According to HE staining results, ISO-pretreated mice obviously alleviated hyperemia and swelling and decreased hepatocellular necrosis in damaged liver (Fig. S2A, B). No significant differences in survival rates were found between ISO-pretreated male and female mice (Fig. S2C, Fig. 1d). Therefore, the gender differences did not appear to play a significant role in influencing the ISO treatment in our models. In addition, previous study has shown there was lack of sexual dimorphism in the response to acute and prolonged isoproterenol-adrenergic stimulation in C57BL/6NCrl mice [20]. Our additional experiment provides preliminary evidence for this viewpoint in a mouse model of liver disease.

### Validations of Proteomic Data Filtering and Quality Control in the Liver Samples

Our subsequent attention was directed towards investigating the safeguarding mechanism of ISO against liver injury induced by LPS/D-GalN. Since earlier research had indicated that the typical receptors of ISO, such as α- and β-adrenergic receptors, were predominantly presented in non-parenchymal liver cells [5], we next primarily investigated how ISO functioned within the stromal cells. TMT labeling proteomic approach was conducted to identify the protein expression pattern extracted from non-parenchymal cells of mouse liver tissues in the control, ALF, and ISO + ALF groups, respectively.

**Fig. 2:**
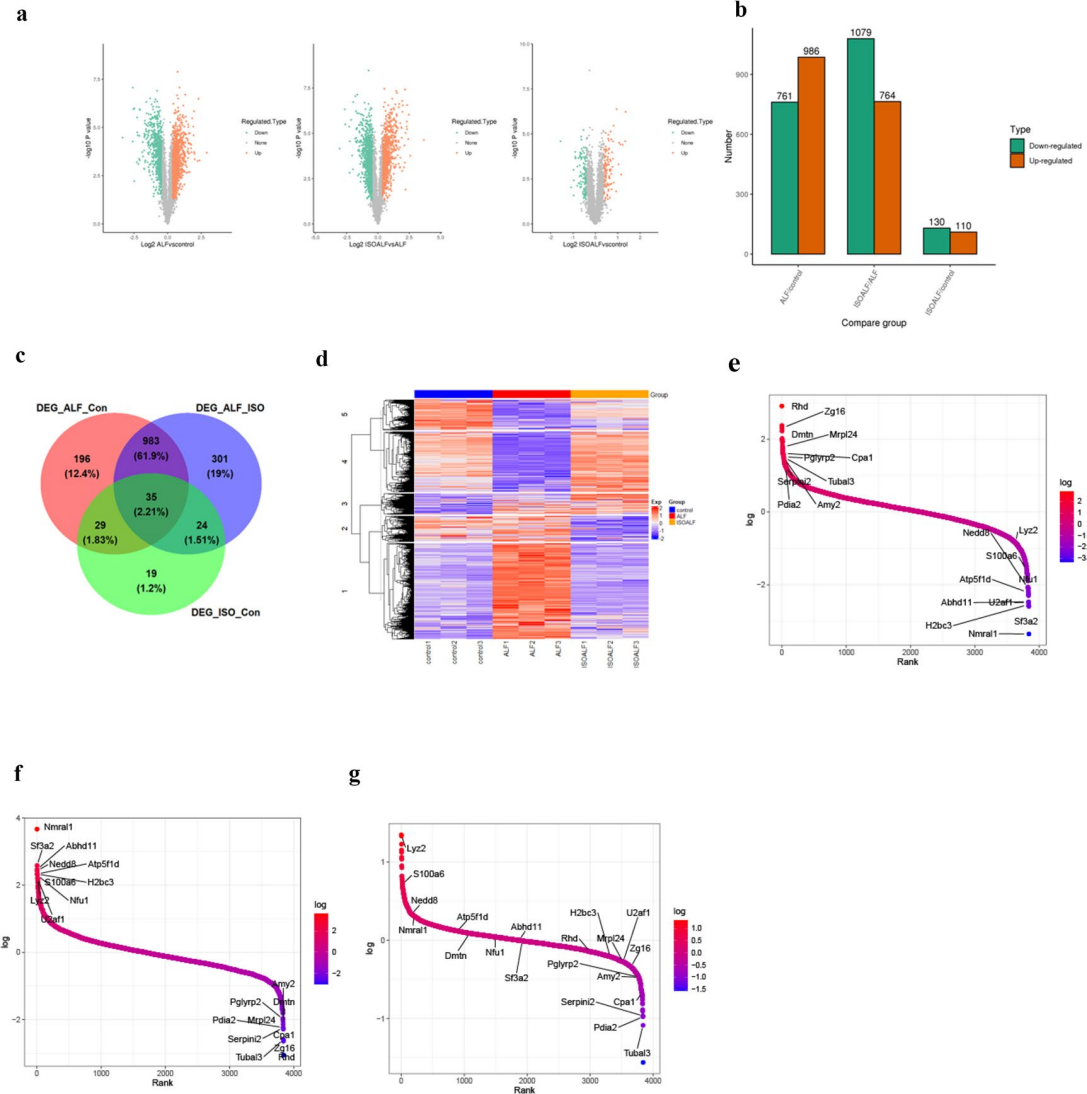
Analysis of differentially expressed proteins (DEPs) in each comparison. **a** Volcano plots showing the fold changes of protein expressions in different comparisons: left: ALF *vs.* Con, middle: ISO + ALF *vs.* ALF, right: ISO + ALF *vs.* Con. **b** Statistical number of upregulated and downregulated DEP profiles in each comparison. **c** Venn diagram showing the interactions of all DEPs analyzed in three compared groups. **d** Hierarchical clustering heatmap analysis of difference in protein expression. Rank abundance plots presented the proteins in each comparison: **e** ALF *vs.* Con, **f** ISO + ALF *vs.* ALF, and **g** ISO + ALF *vs.* Con. The top 10 upregulated and downregulated proteins screened from the comparison of ISO + ALF *vs.* ALF were especially labeled out.

In order to achieve accurate analysis outcomes, the spectrogram, peptide, and protein identification were set with an accuracy FDR of 1% to obtain high-quality results (Fig. 2). A grand total of 320,737 spectrums were generated through the process of mass spectrometry analysis. There was a total of 56,779 spectrums that aligned with the protein, and the spectrograms were utilized at a rate of 17.7%. Spectrogram analysis successfully identified a grand total of 18,297 peptides, with a remarkable count of 17,696 distinct peptide segments. Ultimately, a total of 3914 proteins were identified, of which 3840 could be measurable (Fig. S3A). The verification of data quality control was demonstrated in Fig. S3B–F, which included an analysis of the distribution of protein molecular weight, peptide length, peptide number, and protein coverage. A majority of proteins had a coverage of less than 30% (Fig. S3B), and the mainly peptides were found within the range of 6 to 20 amino acids (Fig. S3C). Figure S1E depicted that the majority of proteins are represented by two or more peptides. When quantifying, it was advantageous for enhancing the precision and reliability of quantitative outcomes to get a protein that corresponds to multiple specific peptides (or multiple spectrograms). Overall, this data confirmed that the outcomes of filtering the data in liver samples met the standards of quality control.

Principal component analysis (PCA) result showed that the data within the group were spatially clustered (Fig. S3F). It displayed clear differences of protein expression pattern between the treated and control groups. The PCA results could annotate 80% of the components in each sample. After normalized the expression data (Fig. S3G), we used method of Pearson correlation coefficients to find that there was a weak correlation between samples from different groups (−0.76 to 0.51; Fig. S3H) but a significant association between the protein expression patterns of each sample in the same group (control group: 0.79–0.80, ALF group: 0.93–0.94, ISO + ALF group: 0.82–0.83, Fig. S3H). Overall, these findings indicated that the quantitative reproducibility of all isolated liver protein samples was satisfactory.

### Identification of Differentially Expressed Proteins Between the ISO Treated and Non-treated ALF Mice

To filter DEPs in each comparison, a volcano plot with a 1.5-fold change cut-off and an adjusted *P* value < 0.01 was used (Fig. 2a). In all, 1587 proteins showed significant expression across several comparisons. LPS/D-GalN treated mice showed 968 upregulated and 761 downregulated differential proteins in comparison to the control group. A comparable quantity of differential epitopes (764 upregulated and 1079 downregulated) was noted between the ISO + LPS/D-GalN treated group and the LPS/D-GalN injected group. Nevertheless, when ISO + LPS/D-GalN was compared to the control group, fewer DEPs (110 upregulated and 130 downregulated) were found (Fig. 2a, b). A Venn diagram was used to further analyze the features of DEPs, and it revealed that 64.1% of DEPs were shared by the comparisons of LPS/D-GalN *vs.* Con and LPS/D-GalN *vs.* ISO + LPS/D-GalN (Fig. 2c). Afterwards, the distinct expression of proteins was organized and displayed in a hierarchical cluster heatmap (Fig. 2d). The findings of clustering analysis showed a closer relationship between ISO + LPS/D-GalN and control group, suggesting that the protein expression patterns were comparable in both groups. In contrast, the clustering heatmap exhibited a clear distinction of ISO + LPS/D-GalN from LPS/D-GalN group, denoting that the expression patterns of differential proteins in ISO-drug-used and healthy groups were quite different from those in the illness group (Fig. 2d). Furthermore, we ranked proteins based on the fold changes and found the top 20 upregulated or/and downregulated proteins were expressed similarly between the comparisons of ALF *vs.* Con (Fig. 2e) and ALF *vs.* ISO (Fig. 2f). These proteins included upregulated Zg16, Dmtn, Mrpl24, Pglyrp2, Cpa1, Amy2, and Serpini2 proteins and downregulated Nmral1, Atp5f1d, H2bc3, Sf3a2, Abhd11, Nedd8, U2af1, and Nfu1 proteins (Fig. 2g).

### Functional Enrichment Analysis of the Screened DEPs

**Fig. 3:**
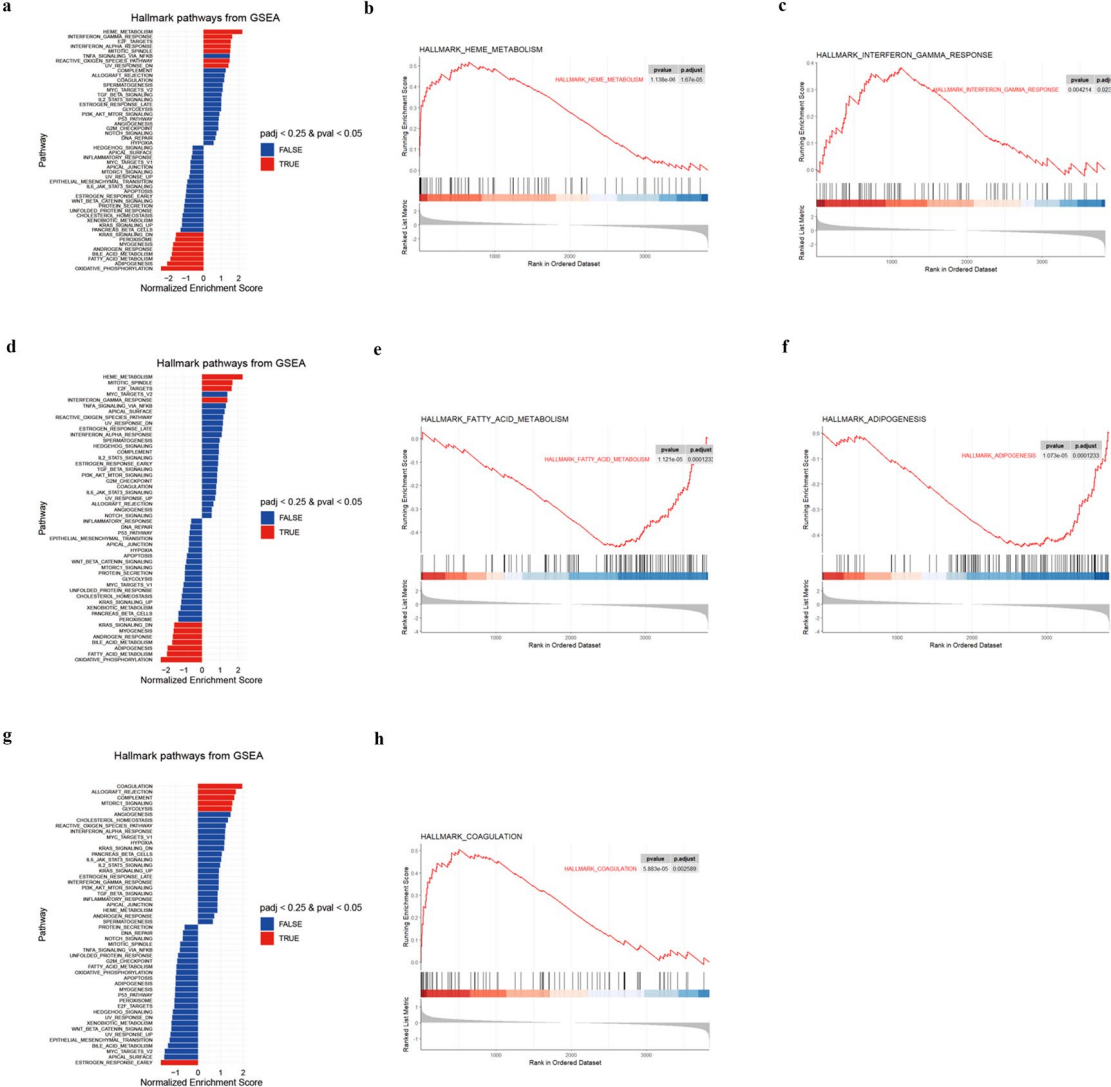
Enrichment pathways with the negative/positive enrichment score after gene set enrichment analysis (GSEA) in different comparisons: **a** ALF *vs.* Con group, **d** ISO + ALF *vs.* ALF group, **g** ISO + ALF *vs.* Con group. Individual GSEA enrichment plots for top enriched categories: **b** Heme metabolism and **c** interferon gamma response in ALF *vs.* Con group, **e** fatty acid metabolism and **f** adipogenesis pathway in ISO + ALF *vs.* ALF group, and **h** coagulation pathway in ISO + ALF *vs.* Con group.

For each comparison, GSEA, GO, and KEGG pathway enrichment analysis were conducted to classify the annotated proteins and ascertain their functions. When comparing the ALF group to the control group, the GSEA results presented in Fig. 3a showed the proteins were positively enriched in heme metabolism (Fig. 3b), interferon gamma response (Fig. 3c), TNFA signaling pathway, and fatty acid metabolism pathway. Heme metabolism, interferon gamma response, fatty acid metabolism (Fig. 3e), and adipogenesis (Fig. 3f) pathway were also enriched in the comparisons of ALF *vs.* ISO + ALF group (Fig. 3d). Coagulation (Fig. 3h), complement, and MTORC1 signaling pathway were enriched in in the comparisons of ISO + ALF *vs.* Con group (Fig. 3g).

**Fig. 4:**
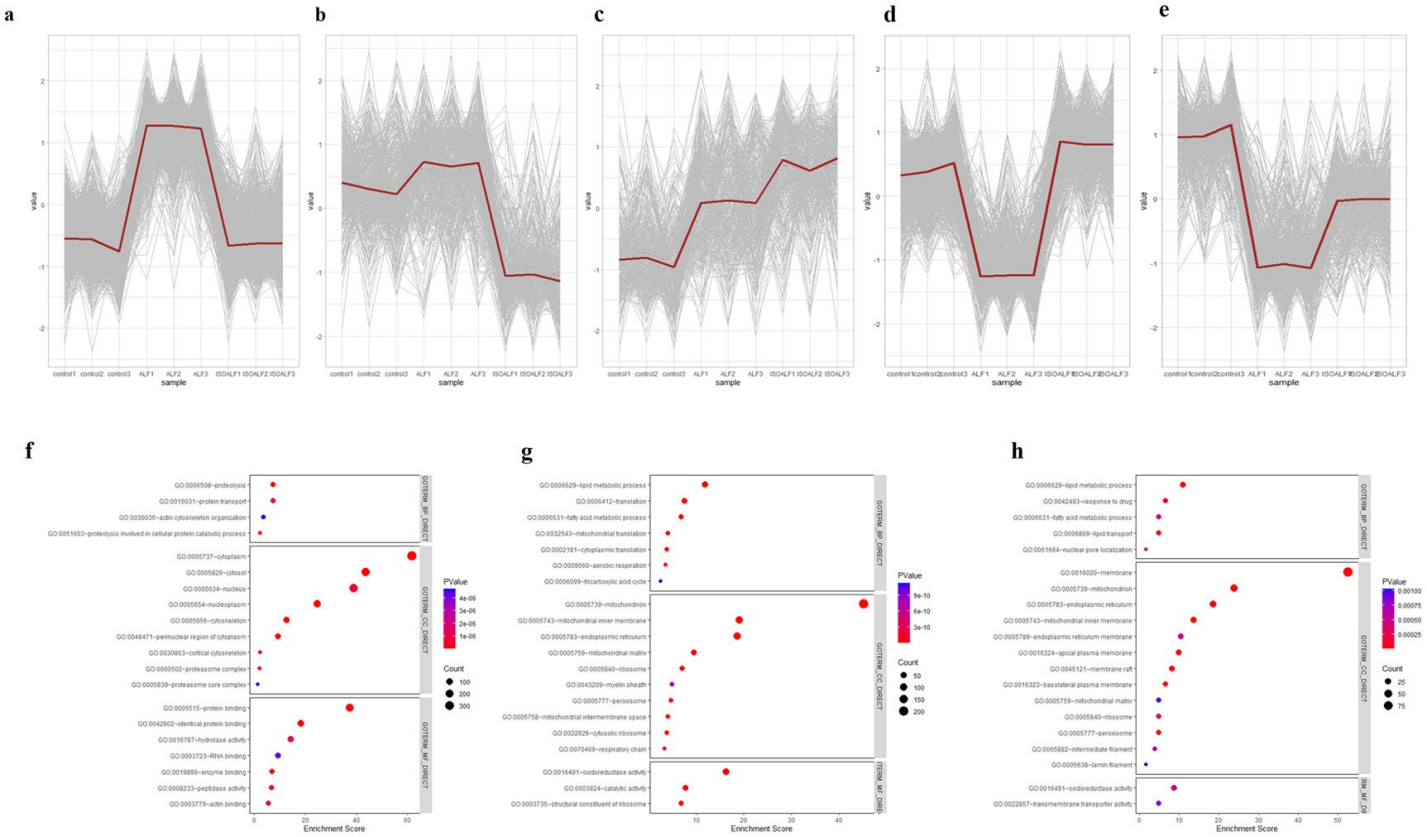
Hierarchical clustering heatmap revealed five distinct temporal patterns of proteins expression identified by K-means clustering. Protein expression changes in **a** Cluster 1, **b** Cluster 2, **c** Cluster 3, **d** Cluster 4, and **e** Cluster 5 across different groups. Bubble diagrams displaying the top 10 items of Gene Ontology (GO) for **f** Cluster 1, **g** Cluster 4, and **h** Cluster 5.

**Fig. 5:**
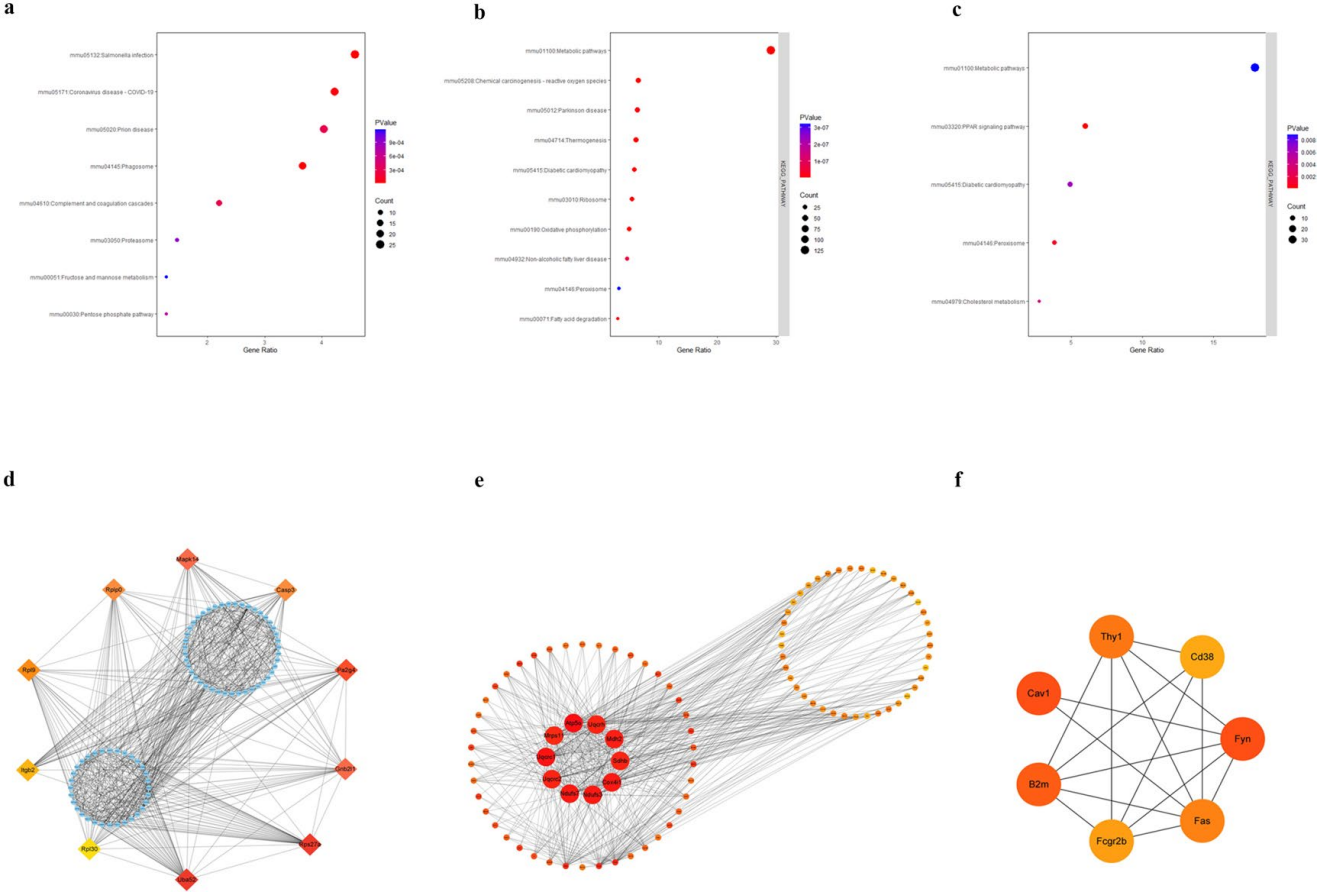
Bubble diagrams displaying the top 10 items Kyoto Encyclopedia of Genes and Genomes (KEGG) pathway enrichment analysis of the three clustering differentially expressed proteins (DEPs): **a** Cluster 1, **b** Cluster 4, and **c** Cluster 5. The interaction network of the hub proteins presented in different clusters for Cluster 1 (**d**), Cluster 4 (**e**), and Cluster 5 (**f**).

To visualize the effect of ISO on anti-acute liver failure, hierarchical cluster analysis was performed with the 3095 DEPs. In order to identify changes in DEPs, we calculated five hierarchical clusters, three of which showed closed features to the state of control group. The clustered DEPs were subjected to GO enrichment analysis to examine their functions using DAVID Functional Annotation Bioinformatics. Cluster 1 proteins were majorly upregulated in the ALF group, of which were enriched in GO terms of proteolysis, cytoplasm, proteasome complex, and protein binding (Fig. 4f). According to the KEGG pathway analysis, it was found that the proteins in cluster 1 were enriched in coronavirus disease, prion disease, and phagosome terms (Fig. 5a). The proteins in cluster 4 were involved in the cell components of mitochondrion and endoplasmic reticulum and in the terms of lipid metabolism, fatty acid metabolism, and oxidoreductase activity, as well as the KEGG terms of metabolic pathways (Fig. 4g and 5b). The proteins in cluster 5 mainly participated in the lipid metabolic process, membrane component, oxidoreductase activity, and PPAR signaling pathway (Fig. 4h and 5c). The core hub proteins involved in KEGG pathways were provided in Supplementary Table 1.

By constructing a PPI network, the hub DEPs particularly related to inflammation response mechanisms in several comparisons were obtained for a global perspective. MODE was used to modularly observe the important DEPs from each cluster on the heatmap by Cytoscape software. The top 10 hub proteins in cluster 1 are presented in Fig. 5d. The upregulation of Mapk14 and Caspase 3 was playing essential roles in the PPI network of clustered 1. Subunits of respiratory complex, including Uqcrc1, Uqcrc2, Uqcrh, Ndufs7, and Ndufs3 proteins, were verified as the top 10 hub DEPs in the downregulated proteins of clustered 4 in ALF groups (Fig. 5e). Besides, the immune-inflammatory proteins, including B2m, Fas, Cav1, Fcgr2b and CD38 in cytoplasm, were detected as the top 10 DEPs in the cluster 5 in ALF mice (Fig. 5f), indicating that a large amount of pro-inflammatory components were depleted during inflammation. Nevertheless, this network requires verification, but it narrowed the pool of Protein-protein interactions that would have helped non-parenchymal liver cells coordinate their response to LPS/D-GalN-induced ALF.

### Verification of the Activation of Macrophages in Mouse Liver

**Fig. 6:**
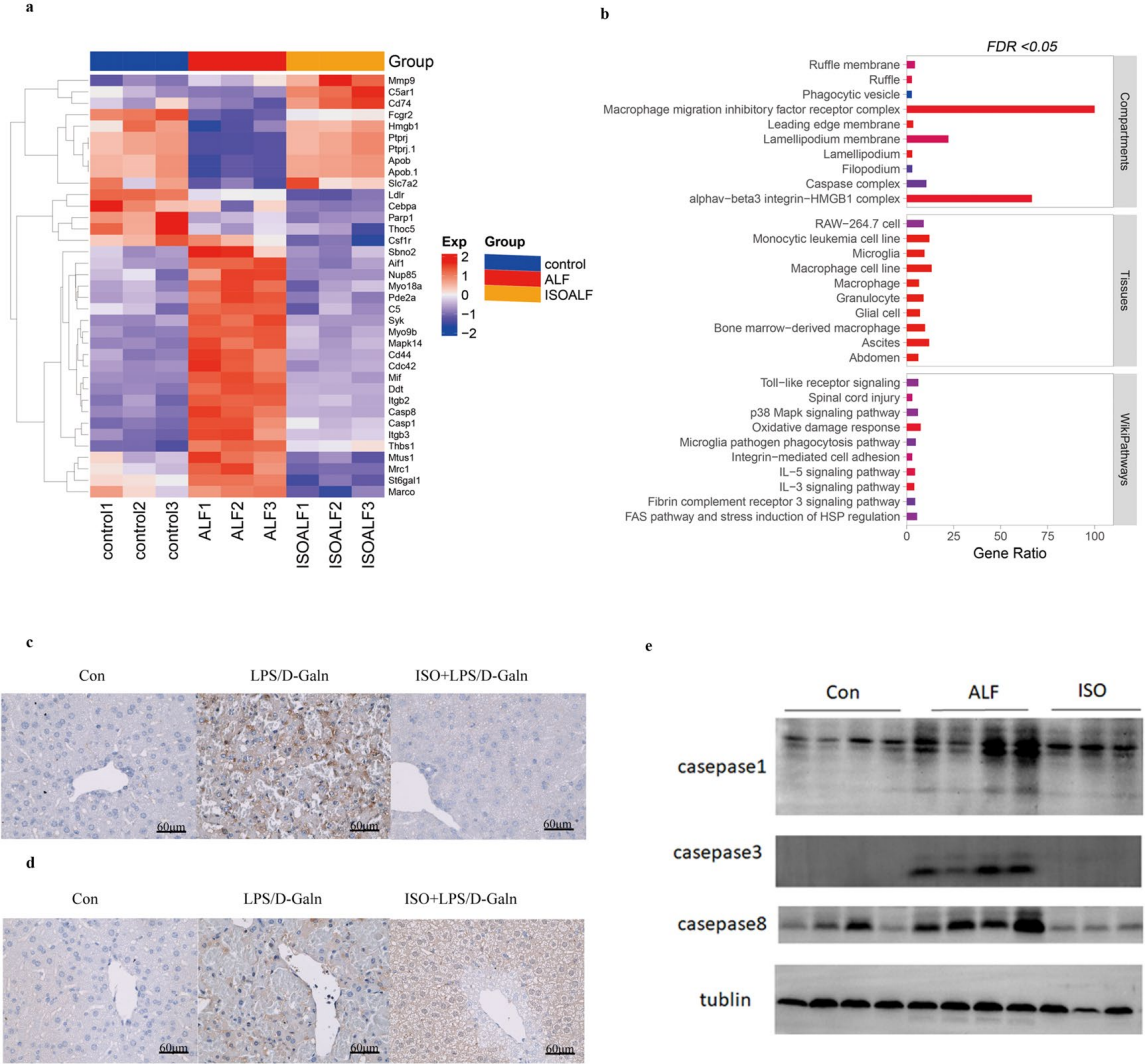
Exploration of macrophage activation in mice liver. **a** Hierarchical clustering heatmap analysis for the absolute quantification levels of DEPs correlated to macrophage activation and **b** bubble diagram displaying the enrichment analysis for cell compartments, tissues, and Wikipathways. Immunohistochemistry stainings of **c** iNOS and **d** Arg-1 for the markers of macrophages in mouse liver tissues. **e** Western blot for the protein expressions of inflammation-induced apoptosis in isolated non-parenchymal tissues.

Since macrophages were the main non-parenchymal cells contributing to inflammatory progression of ALF, we proceeded to identify the features of macrophages in the isolated non-parenchymal cells. First, 29 markers associated with macrophages are chosen and shown in Fig. 6a. The proteomic results showed that the non-parenchymal mouse liver tissues treated with LPS/D-GalN had higher expression levels of Nup85, St6gal1, Myo9b, Mapk14, Marco, Mtus1, Thbs1, Sbno2, Casp1, C5, Ddt, Csf1r, Cd44, Aif1, Casp8, Pde2a, Syk, Mrc1, Itgb2, Mif, Itgb3, and Myo18a proteins compared to the healthy group. Meanwhile, the remaining proteins, Fcgr2, Slc7a2, C5ar1, Apob, Cd74, Hmgb1, and Ptprj, were significantly upregulated in the ISO + ALF group in comparison to the ALF group. The aforementioned 29 DEPs for macrophage markers were further validated by qRT-PCR (Fig. S4). Consistently, the transcript levels of Mapk14, Marco, Mtus1, Thbs1, Sbno2, Casp1, C5, Ddt, Csf1r, Cd44, Aif1, Casp8, Pde2a, Syk, and Mrc1 were upregulated after LPS/D-GalN treatment in comparison with the group that received ISO injection. The expression levels of the coding genes for Apob, Cd74, Hmgb1, and Ptprj were downregulated in a distinct way after the LPS/D-GalN treatment compared to the ISO group, which is consistent with the results of the proteomic study. Nevertheless, the expression levels of the remaining genes contradicted the findings of the proteomic analysis, probably attributed to the impact of multiple process of transcription and post-transcription processes. Subsequently, we imputed these macrophage-related markers into the STRING database and discovered they were belonged to the macrophage migration inhibitory factor receptor, caspase, and HGB1 complex in the cell compartment category, the macrophage cell line in the tissue category, and the inflammatory signaling pathways (Fig. 6b).

In addition, we conducted the IHC assay to identify the pro-(M1) and anti-(M2) inflammation phenotype of macrophage in liver tissues. According to the staining results, it was observed that the M1 macrophage markers, iNOS (Fig. 6c), were more prominent in the non-parenchymal cells of the ALF liver tissues, while the M2 macrophage markers, ARG-1, were more visible in the non-parenchymal cells of the ALF liver tissues after treatment with ISO (Fig. 6d). Ultimately, we conducted Western blotting assay to investigate the expression level of the several hub DEPs (Caspase 8, Caspase3, and Caspase1) in non-parenchymal cells and found these proteins were reduced in ISO-treated ALF mice (Fig. 6f).

### ISO Suppressed Inflammatory Cytokines of THP-1 cell, Probably *via* the Inhibition of p38 MAPK and NF-κB Signaling in THP-1 Cell

**Fig. 7:**
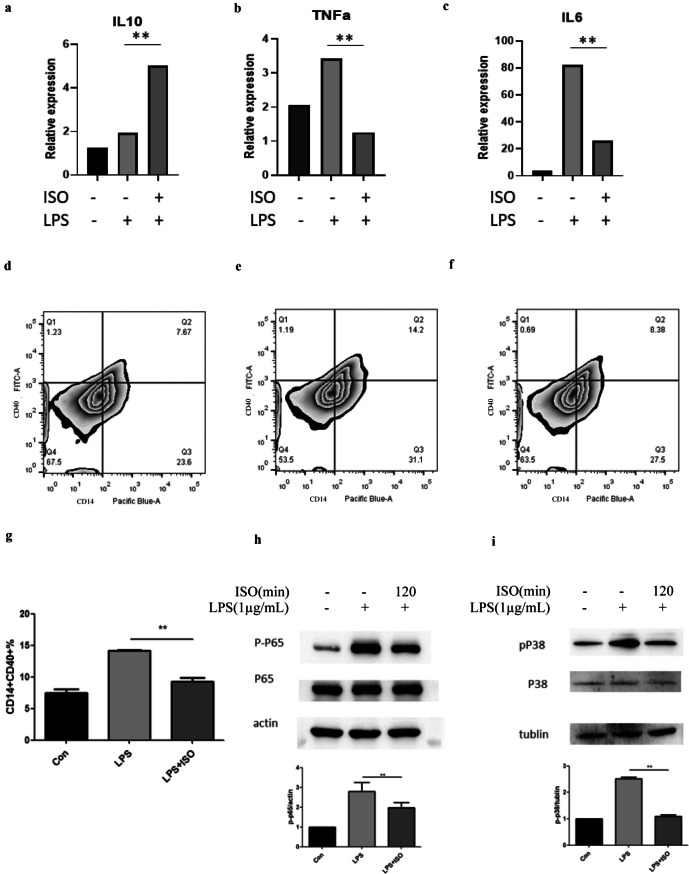
ISO repressed inflammation progress probably *via* p38 and NF-kB signaling in macrophage THP-1 cell line. RT-qPCR for the expressions of IL-10 (**a**), TNF-α (**b**), and IL-6 (**c**) mRNAs in THP-1 cells with LPS (1 µg/mL, 24 h) and ISO (100 nM, pretreated 2 h). Flow cytometry analysis of activation of THP-1 cells among control (**d**), LPS (1 µg/mL, 24 h; **e**), and ISO + LPS (100 nM, pretreated 2 h; then 1 µg/mL, 24 h; **f**) groups with surface antibodies CD14 (Pacific blue) and CD40 (FITC) was calculated with the bar chart (**g**). Western blot for the protein expressions of NF-κB (**h**, upper) and p38 MAPK (**i**, upper) in THP-1 cells was calculated with bar charts in bottom plots. **P < 0.01.

To further verify the proteomic results regarding whether ISO could alter inflammation mechanism through macrophage, we conducted Western blotting assay to investigate the protein expression level of the several hub genes (NF-κB, and Mapk14) *in vitro* THP-1 cell line. The LPS induction markedly upregulated TNF-α, and IL-6 mRNA (*P* < 0.05) excluding IL-10, after 2 h in THP-1 cells; the expression of these cytokine had an available diminish after ISO treatment (*P* < 0.05) (Fig. 7a–c). We conducted flow cytometry to detect the activation of THP-1 cells and stained CD14 and CD40 markers to represent M1 phenotype of macrophages. The population of CD14 + CD40 + macrophages was significantly higher in the LPS treated group (Fig. 7e) than that in the control group (Fig. 7d) and ISO + LPS group (Fig. 7f, g). In order to detect the level of inflammation responses, we performed Western blot assay to investigate the protein expressions of MAPK14 and NF-κB in ISO pretreated THP-1 cells. We found phosphorylation of p38 (Fig. 7h) and NF-kB (Fig. 7i) was significantly higher in the only LPS stimulated group than that in the control and ISO + LPS group, respectively.

In addition, we confirmed the results from the *in vitro* experiment by isolating macrophages from liver tissues. The mRNA extraction revealed that the transcription levels of MAPK14, p50 and p65 were significantly upregulated in the macrophages of the ALF mice, but not in the ISO + ALF mice, as demonstrated in Figs. S4 and S5. This suggests that the MAPK and NF-κB pathway upregulation observed in THP-1 cells has certain reproducibility in *in vivo*, as shown in Fig. 7h and i. Furthermore, the inflammasome marker NLRP3, which is a typical downstream molecule of the MAPK14 and NF-κB pathways, also significantly decreased in ALF mice pretreated with ISO (Fig. S5). This reveals the acute ISO treatment of ALF partially inhibited the inflammatory signaling pathway.

Furthermore, hepatic parenchymal cells were isolated from whole liver tissues to investigate how ISO affects the inflammatory response in hepatocytes. ISO significantly decreased the phosphorylation expression of p38 and p65 in ALF mice (Fig. S6). The expression of total p38 remained unchanged after ISO treatment, whereas the expression of p65 was significantly upregulated. As expected, ISO had an impact on the inflammatory pathways in the hepatic parenchymal cells. It is necessary to carry out additional research to identify the fundamental molecule implicated in the effects of ISO on the hepatocytes.

## Discussion

The injection of LPS/D-GalN is a common approach to create a mouse model of ALF [21, 22]. Agreed with previous models, our study shows that the liver tissue from LPS/D-GalN treated mice exhibited more pronounced inflammation and hyperemia than that in healthy mice, and this was associated with poor survival and liver dysfunction. To identify related molecule mechanisms involved in ALF progression, we isolated proteins from liver non-parenchymal tissue for consequent TMT analysis.

Our present study was the first to demonstrate that the prior administration of SNS agonist, ISO, showed effective prevention against the hepatotoxicity and mortality caused by LPS/D-GalN by decreasing levels of ALT and necrosis. The mechanism underlying the protective effects of ISO appears to be due to shield non-parenchymal hepatocytes from LPS/D-GalN-induced toxicity and inflammation. Additional research needed to prolong the investigation whether a delay administration of ISO still produced protective affection.

ALF is a severe liver injury accompanied acute inflammation, and many efforts have been taken to develop therapies to mitigate its condition. Numerous evidences have suggested that the liver is intricately innervated by the sympathetic nervous system (SNS) to control innate immunity and liver fibrosis. Activation of beta-adrenergic receptors by neurotransmitters has been known to contribute to the regulation of SNS on normal and pathogenesis of liver disease diseased liver function. As a non-selective beta-adrenergic receptor agonist, ISO has been revealed to induce the proliferation of progenitor stem liver cells and alleviate the progression of acetaminophen-induced liver injury [12]. Furthermore, beta-adrenergic receptors were able to accelerate the proliferation of parenchymal hepatocytes to mitigate the development of liver failure. However, during the pathogenesis of acute liver failure, inflammatory mechanisms are be reckoned with and will certainly impact the liver damage. Non-parenchymal liver tissues, such as Kupffer cells, hepatic stellate cells, and endothelial cells, are important components contributing to inflammation. However, no studies have reported the dynamic mechanisms of protein molecules in these non-parenchymal liver cells especially during the ISO treatment of ALF [11]. In our present study, ISO was pre-injected into mice before LPS/GalN induction and protected the mice from acute liver damage, as evident by the morphology of microscopical observations, which effectively reduced the inflammatory invasion and mortality of ALF mice, and was consistent with the impact of ISO in other animal model of ALF. We isolated proteins from non-parenchymal liver cells for TMT proteomic analysis to make a thorough understanding of the protein functions. Quality control of our proteomic data indicated the successful isolation. Interestingly, the amount of protein in the comparison of the ISO *vs.* ALF group was comparable to that in the comparison of the control *vs.* ALF group. Moreover, over 50% of the DEPs in the ISO *vs.* ALF group comparison were shared with the control *vs.* ALF group comparison, and their fold changes had similar tendency. Altogether, these observations confirmed the effect of ISO treatment in alleviating the development of ALF.

Lipopolysaccharide (LPS) is a commonly encountered pathogen-associated molecular pattern that is discharged by Gram-negative bacteria and can stimulate efficacious innate immune responses towards subsequent infections [14, 23]. Recognition of lipopolysaccharide pattern by Toll-like receptor (TLR) contributes to activation of multiple signaling components, *e.g.*, NF-κB and MAPK, and the subsequent production of pro-inflammatory cytokines. The intraperitoneal application of lipopolysaccharide (LPS) combing with other hepatotoxins is a general experimental approach to achieve acute liver injury model [23]. After administering LPS/D-GalN, we screened out the top 10 upregulated and downregulated proteins in non-parenchymal liver cells through comparison with an ISO pretreated mouse model. We then validated the ranking order of these proteins by comparing with control mice. Human zymogen granule protein 16 (ZG16), mitochondrial ribosomal protein large 24 (MRPL24), peptidoglycan recognition protein 2 (PGLYRP2), carboxypeptidase A1 (CPA1), pancreatic alpha-amylase (AMY2), and serpin I2 (SERPINI2) were also highly expressed in LPS/D-GalN mice when compared to the group treated with PBS. ZG16 is highly expressed in mucus-secreting cells, plays a role in pathogen identification by binding to glycosaminoglycans, and has potential functions in promoting local T-cell immunity [24]. Mitochondrial ribosomal protein has been shown to be a contributor to primary deficiencies in mitochondrial respiratory chain activity as well as other inflammatory disorders. Mutations in MRPL24 genes may result in cerebellar atrophy and intellectual disability [25]. PGLYRP2, a protein of host defense highly conserved in mammals that detect bacterial cells, is mainly expressed in the liver [26]. What’s more, PGLYRP2 gene expression was significantly increased in bone marrow-derived macrophages [26]. Carboxypeptidases, a large group of enzymes that cleave one or two amino acids from the carboxy terminus of proteins or peptides, may be involved in antigen processing and may be associated with the production or regulation of several pro-inflammatory mediators [27, 28]. Serpins, also recognized as serine protease inhibitors, utilize a unique and irreversible mechanism differing from typical protease inhibitors. They are linked to inflammatory diseases, including COPD and chronic lung diseases, and exhibit an increase in expression [29]. Consequently, these functions of upregulated proteins in our study inferred that ISO had the capability to suppress pathogen recognition and decrease pro-inflammatory progression of live.

NMRAL1, a redox-sensitive transcriptional regulator, was identified as one of the downregulated proteins in the comparison of ISO *vs.* ALF. Decreased NMRAL1 levels resulted in increased nitric oxide (NO) production and reduced cell viability. Additionally, NMRAL1 has been shown to be a possible mechanism for controlling inflammatory signaling in LPS-treated cells [30]. SF3A2 was one of the subunits SF3A complex that were required for mRNA splicing, and the SF3A mRNA splicing was suspended to inhibit by LPS induced MyD88 [31]. αβ-hydrolase domain-containing 11 (ABHD11), a mitochondrial enzyme that controls OGDHc activity, is the master inhibitor of HIF-1α that plays a crucial role in activating inflammatory macrophage [32, 33]. The mutant U2 small nuclear RNA auxiliary factor 1(U2AF1) leaded to elevated basal mRNA levels of IL-6 and IL-8, but not TNFα, when compared to the U2AF1-wild macrophage [34]. NFU1 iron-sulfur cluster scaffold protein (NFU1) assembles [4Fe-4S] clusters and delivers them to target proteins. Iron–sulfur (Fe–S) clusters are required for numerous biological processes but its biogenesis machinery could be actively suppressed by TLR activation [35, 36]. Therefore, according to the biological functions of these downregulated proteins, we can suspend ISO certainly increased the anti-inflammation mechanism in hepatic non-parenchymal tissues damaged by LPS/D-GalN.

When we submitted the differentially expressed proteins (DEPs) for functional enrichment analysis in the David dataset, we observed upregulation of hub proteins associated with COVID-19 pathway and/or prion disease (RPL30, RPL12, C1QA, C1QB, STAT3, MAPK14, CASP1, SOD1, CASP3) in cases of acute liver damage [37, 38]. The alpha-subunit (PSMA) and the beta-subunit (PSMB) of the 20S proteasome complex in the KEGG pathway of prion disease had been identified as LPS-binding proteins [39, 40], which was comprehensible for their upregulations in LPS/D-GalN-induced liver inflammation. Conversely, these proteins were alleviated in cases of ISO pretreated acute liver failure. Especially, the expression of pro-inflammatory proteins of STAT3, MAPK14, CASP1, SOD1 and CASP3, correlated to the activation of M1 macrophage [41], was increased in ALF. It was expected that the core molecules related to COVID-19 pathway and prion disease would also be upregulated in the ALF model. Our *in vivo* and *in vitro* assays confirm that acute ISO stimulation reduced inflammatory response in non-parenchymal cells of ALF liver tissues, particularly in macrophages.

Following acute liver injury caused by LPS/D-GalN, the metabolic pathway was severely damaged, resulting in the disruption of the mitochondrial respiratory chain. The family members of cytochrome c oxidase (COX) and NADH dehydrogenase (complex I) are crucial complex proteins in the mitochondrial respiratory chain [42, 43]. Patients with acute liver failure were found to have combined respiratory chain deficiency and reduced numbers of mitochondria [43]. Furthermore, mitochondria are essential for the modulation of macrophage function. Indeed, macrophages stimulated with lipopolysaccharide (LPS) showed a rewired metabolism manifested by decreased mitochondrial respiration, inhibition of the TCA cycle, and upregulation of aerobic glycolysis [44]. Thereafter, in terms of the mechanism, inhibited NAD + and the NAD + -dependent signaling pathway of metabolism contributed to liver damage in ALF [45]. Enhancing mitochondrial function within the metabolic pathway could be targeted as part of a multifaceted therapeutic approach for various forms of liver disease. Furthermore, for the activation of peroxisome proliferator-activated receptor α (PPARα) pathway, which is a ligand-activated transcription factor that controlled lipid metabolism, it plays an important role in safeguarding the liver and reducing hepatocyte apoptosis in cases of ALF [46].

Acute liver failure is a systemic inflammatory response comprising pro-inflammatory and anti-inflammatory components. It has been observed that CD38 deficiency may promote inflammation by activating Sirt1/NF-κB in macrophages [47]. In patients with ALF, anti-inflammatory activity mediated by FcγRIIB-associated IgG1 is defective [48]. Furthermore, Caveolin-1 negatively regulates inflammation by attenuating vascular injury caused by Low-Density Lipoprotein [49, 50]. Fyn plays a role in the neurite outgrowth induced by angiotensin II type 2 receptors [51]. Tyrosine kinase Fyn regulates iNOS expression stimulated by LPS [52]. It is evident that certain pro-inflammatory molecules were rapidly exhausted and accompanied by a lack of anti-inflammatory mechanisms after LPS stress [53, 54]. Thereafter, ISO may also be involved in regulating the systemic inflammatory response during acute liver injury caused by LPS/D-GalN in our present study.

Heme metabolism, fatty acid metabolism, and mitochondrial oxidation signaling pathways are crucial in anti-inflammatory macrophage polarization [55–57]. ISO potentially affects the progression of ALF by regulating fatty acid metabolism and mitochondrial oxidation in monocytes/macrophages and promoting the upregulation of anti-inflammatory molecules to reduce ALF severity. Consequently, additional research must be conducted to ascertain the exact function of ISO in reducing ALF inflammation and whether this is attributed to a biological response from other liver cells rather than a direct impact on macrophages.

From the above discussion, it appears that isoproterenol plays a different role in acute liver injury than it does in inducing myocardial injury [58]. A large amount of studies have shown that ISO causes a significant increase in inflammation in the field of cardia research. Therefore, we required to investigate whether isoproterenol had a different function in regulating macrophage activity in acute liver damage compared to the role in cardiac macrophages [59].

Activated macrophages have two different phenotypes, namely M1 (classically activated) and M2 (alternatively activated), which are attributed to different stimuli. M1 macrophages are pro-inflammatory, essential in the host defense mechanism, while M2 are related to responses involving anti-inflammatory reactions and tissue remodeling [41]. Since macrophages are the key cells involved in the systemic inflammation of ALF, we conducted an analysis of the 29 DEPs (differentially expressed proteins) associated with macrophage functions. Results from the PPI analysis using the STRING database showed that these DEPs were mostly enriched in macrophages. The proteins related to M1 macrophages were significantly decreased in the ISO-treated group, while the DEPs related to M2 macrophages were statistically increased. RT-qPCR confirmed the mRNA levels in the animal models. We further studied the effects of ISO treatment on inflammation regulation in a human macrophage cell line. Previous studies have shown that the β-adrenergic receptor agonist, isoproterenol, synergistically amplifies the anti-inflammatory role of cAMP pathway inducers, resulting in reduced expression of the key pro-inflammatory cytokine TNFα, and increased expression of the key anti-inflammatory cytokine IL-10 in macrophage cell lines [60, 61]. Our results were consistent with this, showing that ISO could effectively reduce inflammation in ALF mice.

Currently, a probable explanation for the clear evidence of ISO’s protective effect on the liver is that the expansion of hepatic precursor cells leads to the release of Wnt ligands. These ligands safeguard hepatocytes from impairments [11]. Nonetheless, it necessitates a reasonably prolonged duration to proliferate sufficient fresh hepatocytes from liver progenitor cells following acute ISO administration to obstruct the progression of damage. Other potential mechanisms, such as the metabolic and inflammation pathways, could contribute to a rapid alteration in biological functions under ISO treatment to limit acute liver hepatotoxicity. Previous studies have indicated the possible influence of the sympathetic nervous system on the immune response and associated inflammation [62–64]. Thus, it is plausible that ISO treatment partially protects the liver by modulating the inflammatory process. Previous research indicates that the hepatic sympathetic nervous system plays a crucial role in preventing Fas-induced hepatic injury by means of antiapoptotic proteins [27] and IL-6 [65]. The observation of a significant difference in IL-6 expression following previous ISO treatment further supports our model outcomes.

However, we must acknowledge the limitations of our study. Although the proteomic data displayed a widespread impact of ISO on non-parenchymal cells, further analysis is necessary to determine its particular effects on specific types of these cells. Furthermore, more precise studies are required to comprehensively understand how ISO controls macrophage phenotype in Kupffer cells.

## Conclusion

In conclusion, our study presents the first comprehensive quantitative proteomic analysis of freshly isolated non-parenchymal hepatocytes to investigate the impact of SNS stimulation on ALF. The protection of the liver against acute injury by non-parenchymal hepatocytes is mediated by several pathways. Our findings highlight that early administration of ISO may be a potentially beneficial treatment for LPS/D-GalN-induced hepatotoxicity, given its efficacy, safety, and easy accessibility.

### Supplementary Information

Below is the link to the electronic supplementary material.

Supplementary Table 1 List of hub genes involve in KEGG pathways (DOCX 19 KB)

Figure S1. Safety of acute ISO injection. Different doses of ISO with 0, 10mg/kg and 100mg/kg were given for 6h in mice, respectively. (A) Morphological observation of liver tissue. (B) Representative sections of hematoxylin-eosin (H&E) staining (100-fold) in liver tissues. (C) Serum levels of alanine transaminase (ALT), and aspartate transaminase (AST). Statistical difference was performed by Student’s t-test in each comparison. n.s represented P >0.05. (PNG 6242 KB)

Figure S2. Effect of ISO treatment in male ALF mice. (A) Morphological observation of liver tissue. (B) Representative sections of hematoxylin-eosin (H&E) staining (100-fold) in liver tissues. (C) Survival curves in different group of mice. (PNG 647 KB)

Figure S3. Quantitative of screened proteins and data quality control of liver samples. The number of filtered proteins matched in the search library (A), distribution of protein coverage (B), peptide length (C), protein molecular weight (D), and (E) peptide number per protein. (F) Principal component analysis (PCA) of proteome data among three groups of samples. (G) Normalization of proteomic data, and (H) Pearson correlation analysis of the samples among three groups. (JPG 651 KB)

Figure S4. RT-qPCR analysis for the mRNA expression of 29 differentially expressed protein coding genes related to macrophage activation from isolated liver non-parenchymal tissues. Data are presented as mean ± SEM. Statistical significance was performed by Student’s t-test. *P < 0.05; **P < 0.01; ***P < 0.001. (JPG 180 KB)

Figure S5. RT-qPCR analysis for the mRNA expression of hub proteins related to MAPK14 and NF-κB pathways in isolated liver non-parenchymal cells. Relative mRNA expression of p65 (A), p50 (B) and NLRP3 (C). Data are presented as mean ± SEM. Statistical significance was performed by Student’s t-test between two groups. **P < 0.01. (PNG 68 KB)

Figure S6. Detection of hepatocyte inflammation in mice liver. (A) Western blot for the protein expressions of inflammation signaling pathways in isolated parenchymal liver cells. The relative expression of phosphorylated p38 (B) and phosphorylated p65 (C) were calculated with bar charts. *P < 0.05; **P < 0.01. (JPG 635 KB)

## Data Availability

The datasets generated and/or analyzed during the current study are available from the corresponding author on reasonable request.
