# Supplementary material for: Treatment with β-Adrenoceptor Agonist Isoproterenol Reduces Non-parenchymal Cell Responses in LPS/D-GalN-Induced Liver Injury
Source: Inflammation. 2023 Dec 21;47(2):733–52. doi: 10.1007/s10753-023-01941-z (PMC11074027; doi:10.1007/s10753-023-01941-z)
Supplement: Supplementary file 1 — Supplementary Table 1 List of hub genes involve in KEGG pathways (DOCX 19 KB) [file 10753_2023_1941_MOESM1_ESM.docx]

**Supplementary Table 1 List of hub genes involve in KEGG pathways**

| **KEGG_PATHWAYS for Cluster 1 proteins** | | | |  |
| --- | --- | --- | --- | --- |
| Term | Count | PValue | Genes | FDR |
| mmu04145:Phagosome | 20 | 1.23E-05 | ATP6V1A, DYNC1I2, C1RA, ATP6AP1, NCF1, TUBAL3, NCF2, ITGB3, NCF4, ITGB2, TUBB4B, THBS1, TUBA4A, DYNC1LI1, TUBB6, LAMP1, CD14, ATP6V1E1, ATP6V1D, ATP6V0A1 | 0.001669 |
| mmu05171:Coronavirus disease - COVID-19 | 23 | 3.42E-05 | C1QB, C1QA, RPL30, C1RA, VWF, RPL12, STAT2, STAT3, RPLP0, F13A1, RPS27L, ADAR, C8B, MAPK14, RPL9, RPS15, RPS25, CASP1, RPLP2, MASP2, RPL29, RPS27A, UBA52 | 0.003098 |
| mmu04610:Complement and coagulation cascades | 12 | 2.90E-04 | C1QB, C1QA, C1RA, VWF, CFH, PROS1, SERPINF2, ITGB2, F13A1, MASP2, C8B, KLKB1 | 0.016096 |
| mmu05020:Prion disease | 22 | 2.96E-04 | C1QB, C1QA, NCF1, TUBAL3, NCF2, NCF4, HSPA2, C8B, MAPK14, TUBB4B, TUBA4A, PSMA7, SOD1, PSMB6, PSMB7, PSMD9, PPP3CA, PSMB4, TUBB6, PSMA3, PSMA4, CASP3 | 0.016096 |
| mmu00030:Pentose phosphate pathway | 7 | 7.38E-04 | RPIA, RPE, G6PDX, PGM2, ALDOC, PFKM, PFKP | 0.033444 |
| mmu03050:Proteasome | 8 | 9.41E-04 | PSMB6, PSMB7, PSMD9, PSMB4, PSMA3, PSMA4, PSMA7, PSMB9 | 0.036555 |
| **KEGG_PATHWAYS for Cluster 4 proteins** | | | | |
| mmu01100:Metabolic pathways | 133 | 1.60E-30 | DMGDH, VKORC1L1, NDUFA13, ALDH1L1, ACAA2, ACSM3, PANK1, NDUFA10, CPOX, ABAT, PIP4K2C, HADH, FADS1, GAMT, SDS, ALG5, HOGA1, SDHB, PISD, ALDH3A2, DPM3, ETHE1, UQCRC1, SUCLG2, MT-CO3, UQCRC2, ALDH7A1, LIPT1, GCDH, UQCRB, MAOA, GSTT3, COX7A2, AK4, COX7A1, FHIT, UQCRH, PGS1, ADH1, PNPO, COX11, HAO1, PRODH, HAO2, ARSB, ATP6V1F, PDHA1, IDH2, GOT2, DHCR24, COQ6, COQ5, CS, AMACR, B4GAT1, CPS1, PAH, CYP1A2, CYCS, PLCH1, ADA, ACADVL, HIBADH, COX4I1, TECR, QPRT, HEXA, KEG1, GPT, DHTKD1, AKR1B7, ACADL, ALDH2, AUH, GNPNAT1, ACP5, SCD1, GUSB, ACADS, IDH3A, VKORC1, URAH, PCYT1A, UGT1A1, URAD, GPX7, AMPD2, PRODH2, DDOST, TST, NDUFS7, BDH1, NDUFS5, CHDH, CAT, CYP2C40, NDUFS3, HAGH, ASPA, MVK, NDUFB4, NDUFB3, HSD17B4, PDHB, COX5B, CYP7A1, HMGCL, ALDH1B1, MAT2A, CBR4, NDUFA8, CKM, MDH2, IDH3G, NDUFA4, MMAB, PTGES2, GALNT1, NDUFA1, GATB, FMO3, FMO4, SUOX, ASS1, GNPDA1, GSTA3, GSTA2, CTH, SARDH, NAGS, SCLY, OTC, UOX | 3.96E-28 |
| mmu05208:Chemical carcinogenesis - reactive oxygen species | 30 | 3.47E-11 | NDUFA13, UQCRB, NDUFB4, COX4I1, NDUFA10, NDUFB3, GSTT3, ARAF, COX7A2, PTPRJ, COX5B, COX7A1, UQCRH, HRAS, NDUFA8, NDUFA4, NDUFA1, SDHB, NDUFS7, GSTA3, NDUFS5, GSTA2, CAT, CYP1A2, UQCRC1, NDUFS3, KRAS, MT-CO3, UQCRC2, SLC25A5 | 4.30E-09 |
| mmu00190:Oxidative phosphorylation | 23 | 1.22E-10 | NDUFA8, NDUFA13, UQCRB, NDUFA4, NDUFB4, COX4I1, NDUFA10, NDUFB3, NDUFA1, COX7A2, COX5B, SDHB, COX7A1, UQCRH, NDUFS7, NDUFS5, UQCRC1, NDUFS3, CYCS, COX11, MT-CO3, UQCRC2, ATP6V1F | 1.01E-08 |
| mmu03010:Ribosome | 25 | 1.09E-09 | RPL4, RPL32, MRPS11, MRPL18, RPL11, MRPL17, MRPL15, RPL10A, MRPL20, RPS19, RPL13, RPS2, RPL18, RPS13, RPS12, RPL22, MRPS21, RPSA, MRPS6, MRPS5, RPS27, RPL24, RPS20, RPL26, RPS23 | 6.79E-08 |
| mmu05415:Diabetic cardiomyopathy | 27 | 1.38E-09 | NDUFA13, UQCRB, NDUFB4, COX4I1, NDUFA10, NDUFB3, COX7A2, ATP2A1, PDHB, COX5B, COX7A1, UQCRH, CPT2, MPC1, NDUFA8, PDHA1, NDUFA4, NDUFA1, SDHB, COL1A1, NDUFS7, NDUFS5, UQCRC1, NDUFS3, MT-CO3, UQCRC2, SLC25A5 | 6.86E-08 |
| mmu04714:Thermogenesis | 28 | 2.15E-09 | NDUFA13, UQCRB, NDUFB4, COX4I1, NDUFA10, NDUFB3, COX7A2, COX5B, COX7A1, UQCRH, CPT2, COX11, COA7, HRAS, NDUFA8, NDUFA4, NDUFA1, SDHB, NDUFS7, RHEB, NDUFS5, UQCRC1, GNAS, NDUFS3, NDUFAF3, KRAS, MT-CO3, UQCRC2 | 8.87E-08 |
| mmu00071:Fatty acid degradation | 14 | 3.82E-09 | GCDH, ACADVL, ACAA2, ECI1, ECI2, ALDH3A2, ADH1, CPT2, ACADL, ALDH2, ALDH1B1, HADH, ALDH7A1, ACADS | 1.35E-07 |
| mmu05012:Parkinson disease | 29 | 9.59E-09 | NDUFA13, UQCRB, MAOA, NDUFB4, COX4I1, NDUFA10, NDUFB3, COX7A2, COX5B, COX7A1, UQCRH, TXN2, PSMD8, NDUFA8, TRAP1, HSPA5, NDUFA4, NDUFA1, TUBB4A, SDHB, NDUFS7, NDUFS5, UQCRC1, GNAS, NDUFS3, CYCS, MT-CO3, UQCRC2, SLC25A5 | 2.97E-07 |
| mmu04932:Non-alcoholic fatty liver disease | 21 | 6.08E-08 | NDUFA8, NDUFA13, UQCRB, NDUFA4, NDUFB4, COX4I1, NDUFA10, NDUFB3, NDUFA1, COX7A2, COX5B, SDHB, COX7A1, UQCRH, NDUFS7, NDUFS5, UQCRC1, NDUFS3, CYCS, MT-CO3, UQCRC2 | 1.68E-06 |
| mmu04146:Peroxisome | 15 | 3.21E-07 | PECR, MVK, ECI2, IDH2, HSD17B4, PEX7, GNPAT, HMGCL, AMACR, PRDX1, CAT, HAO1, PEX11G, DECR2, HAO2 | 7.97E-06 |
| **KEGG_PATHWAYS for Cluster 5 proteins** | | | | |
| Term | Count | PValue | Genes | FDR |
| mmu04146:Peroxisome | 7 | 8.96E-04 | PEX16, PHYH, SCP2, ACOX1, EHHADH, PEX11A, CROT | 0.09367 |
| mmu03320:PPAR signaling pathway | 11 | 2.18E-07 | FADS2, SCP2, ACOX1, EHHADH, CYP4A10, PLIN4, PLIN2, CYP4A14, HMGCS2, CD36, PLIN5 | 4.56E-05 |
| mmu04979:Cholesterol metabolism | 5 | 0.004067 | ABCG5, APOC2, VDAC3, LCAT, CD36 | 0.283307 |
| mmu05415:Diabetic cardiomyopathy | 9 | 0.006458 | NDUFA9, NDUFB9, NDUFAB1, PDK4, VDAC3, SDHC, CD36, SDHD, SLC25A4 | 0.337425 |
| mmu01100:Metabolic pathways | 33 | 0.008894 | NDUFB9, ISYNA1, OAT, H6PD, MGST1, GLYAT, FADS2, INPP5A, SMPD4, TDO2, SCP2, ANPEP, RDH16, CD38, HMGCS2, KDSR, NDUFA9, CYP2J5, GPX3, ENTPD5, CYP4A10, SDHC, ALG12, MTHFS, CYP4A14, SDHD, CYP2A5, TMLHE, ACOX1, EHHADH, NDUFAB1, PLPP1, ACOT3 | 0.340897 |
