# Supplementary figures and images for: Treatment with β-Adrenoceptor Agonist Isoproterenol Reduces Non-parenchymal Cell Responses in LPS/D-GalN-Induced Liver Injury
Source: Inflammation. 2023 Dec 21;47(2):733–52. doi: 10.1007/s10753-023-01941-z (PMC11074027; doi:10.1007/s10753-023-01941-z)

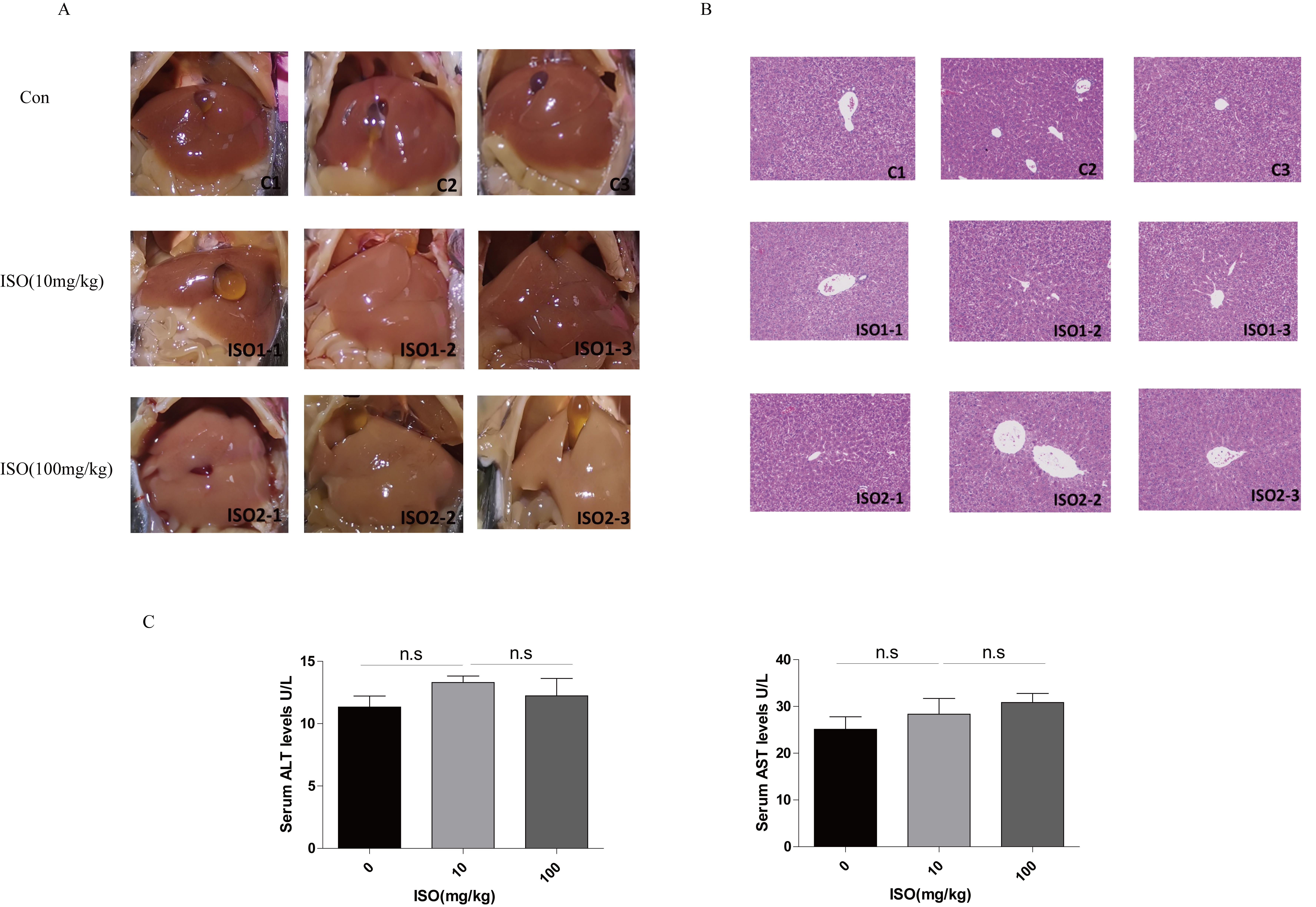

Supplement: Supplementary file 2 — Figure S1. Safety of acute ISO injection. Different doses of ISO with 0, 10mg/kg and 100mg/kg were given for 6h in mice, respectively. (A) Morphological observation of liver tissue. (B) Representative sections of hematoxylin-eosin (H&E) staining (100-fold) in liver tissues. (C) Serum levels of alanine transaminase (ALT), and aspartate transaminase (AST). Statistical difference was performed by Student’s t-test in each comparison. n.s represented P >0.05. (PNG 6242 KB) [file 10753_2023_1941_MOESM2_ESM.png]

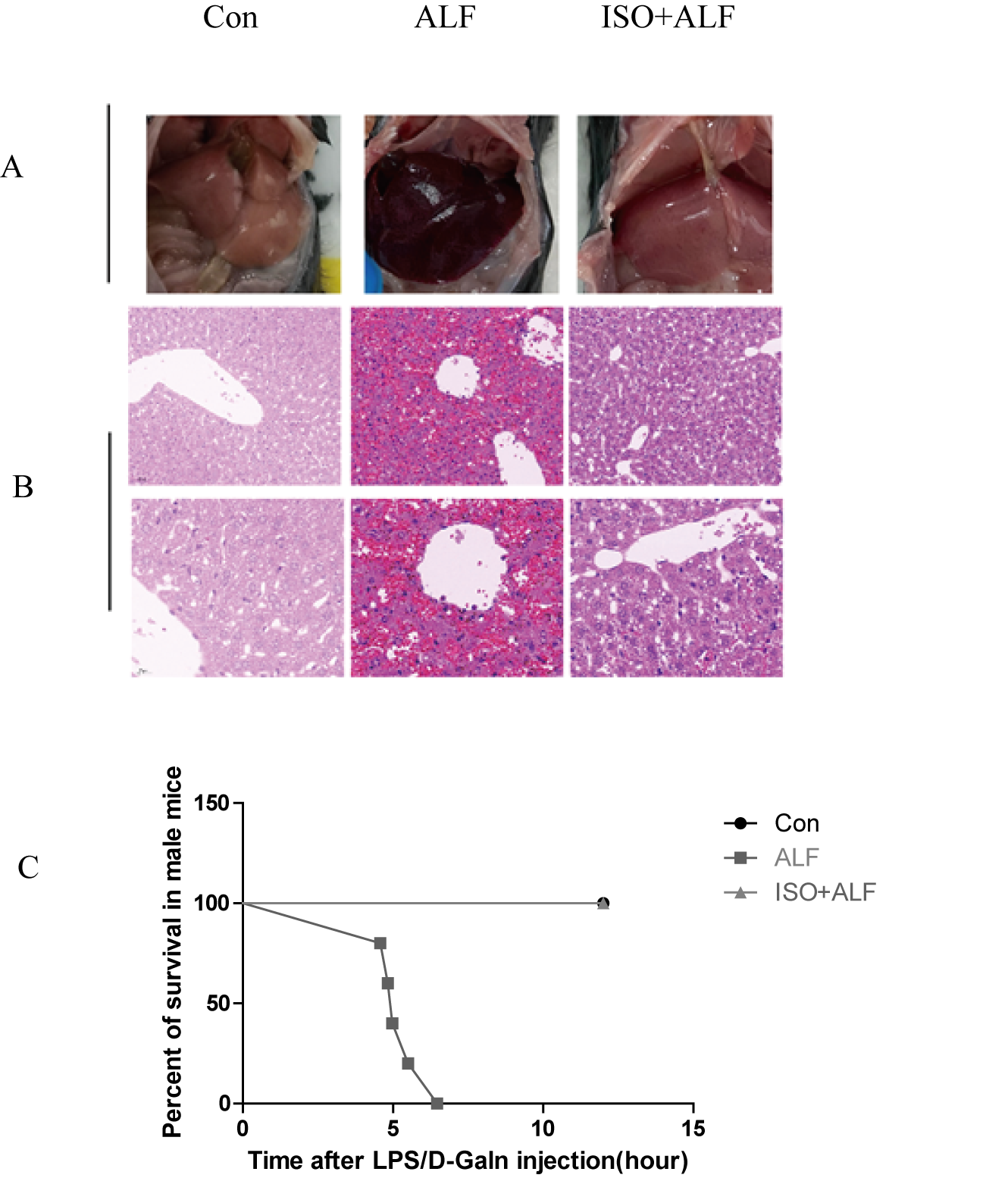

Supplement: Supplementary file 3 — Figure S2. Effect of ISO treatment in male ALF mice. (A) Morphological observation of liver tissue. (B) Representative sections of hematoxylin-eosin (H&E) staining (100-fold) in liver tissues. (C) Survival curves in different group of mice. (PNG 647 KB) [file 10753_2023_1941_MOESM3_ESM.png]

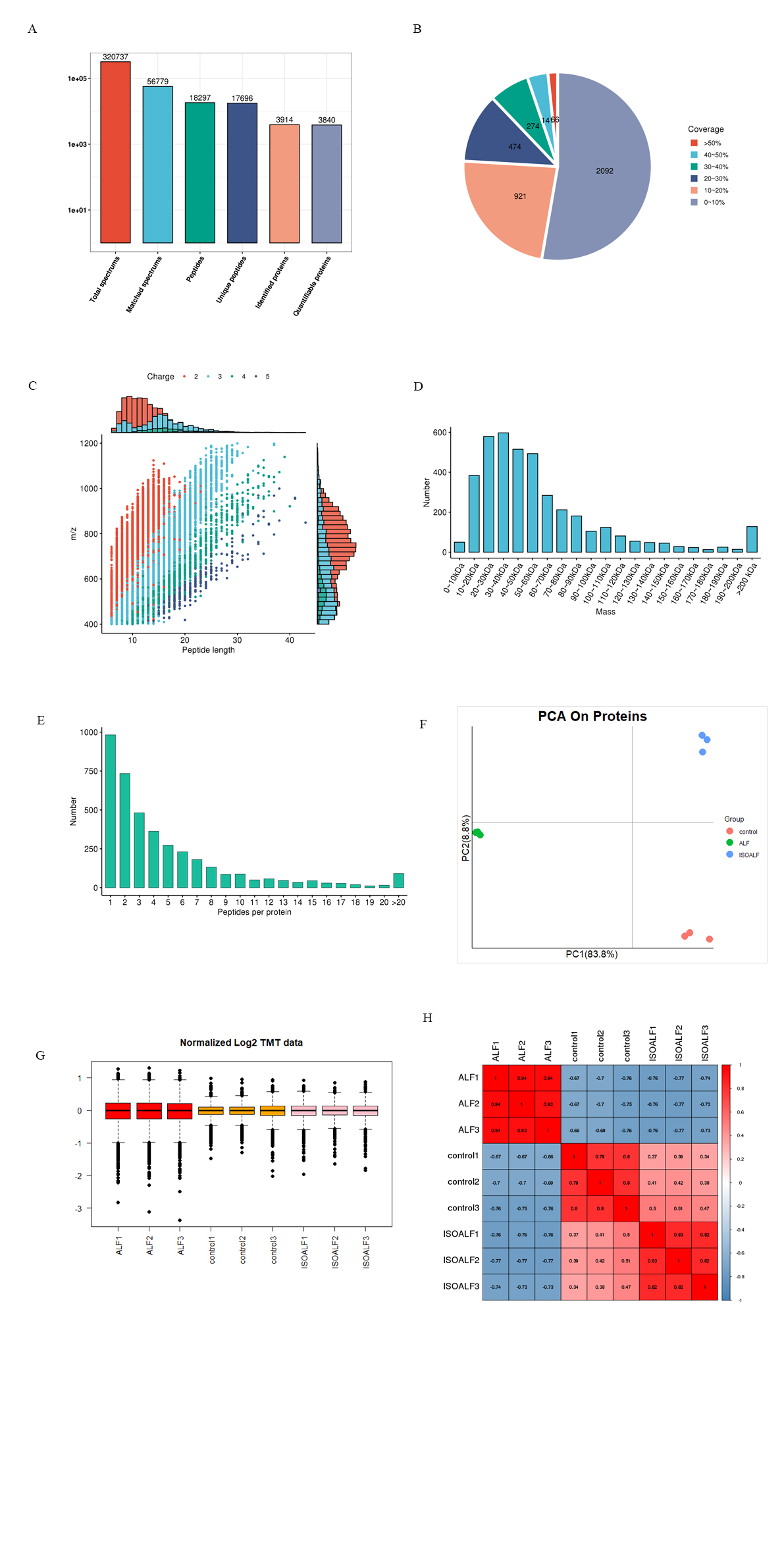

Supplement: Supplementary file 4 — Figure S3. Quantitative of screened proteins and data quality control of liver samples. The number of filtered proteins matched in the search library (A), distribution of protein coverage (B), peptide length (C), protein molecular weight (D), and (E) peptide number per protein. (F) Principal component analysis (PCA) of proteome data among three groups of samples. (G) Normalization of proteomic data, and (H) Pearson correlation analysis of the samples among three groups. (JPG 651 KB) [file 10753_2023_1941_MOESM4_ESM.jpg]

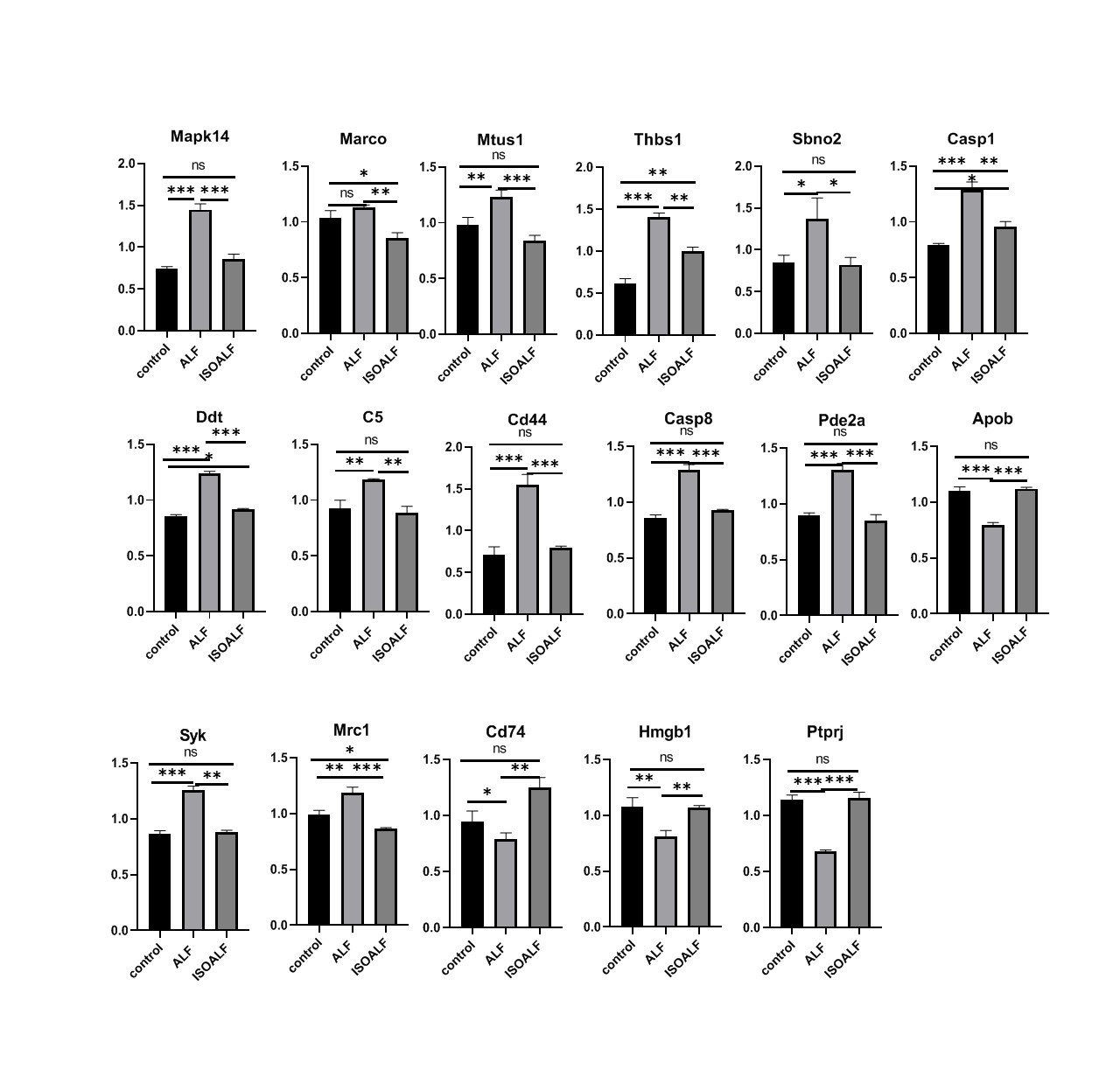

Supplement: Supplementary file 5 — Figure S4. RT-qPCR analysis for the mRNA expression of 29 differentially expressed protein coding genes related to macrophage activation from isolated liver non-parenchymal tissues. Data are presented as mean ± SEM. Statistical significance was performed by Student’s t-test. *P < 0.05; **P < 0.01; ***P < 0.001. (JPG 180 KB) [file 10753_2023_1941_MOESM5_ESM.jpg]

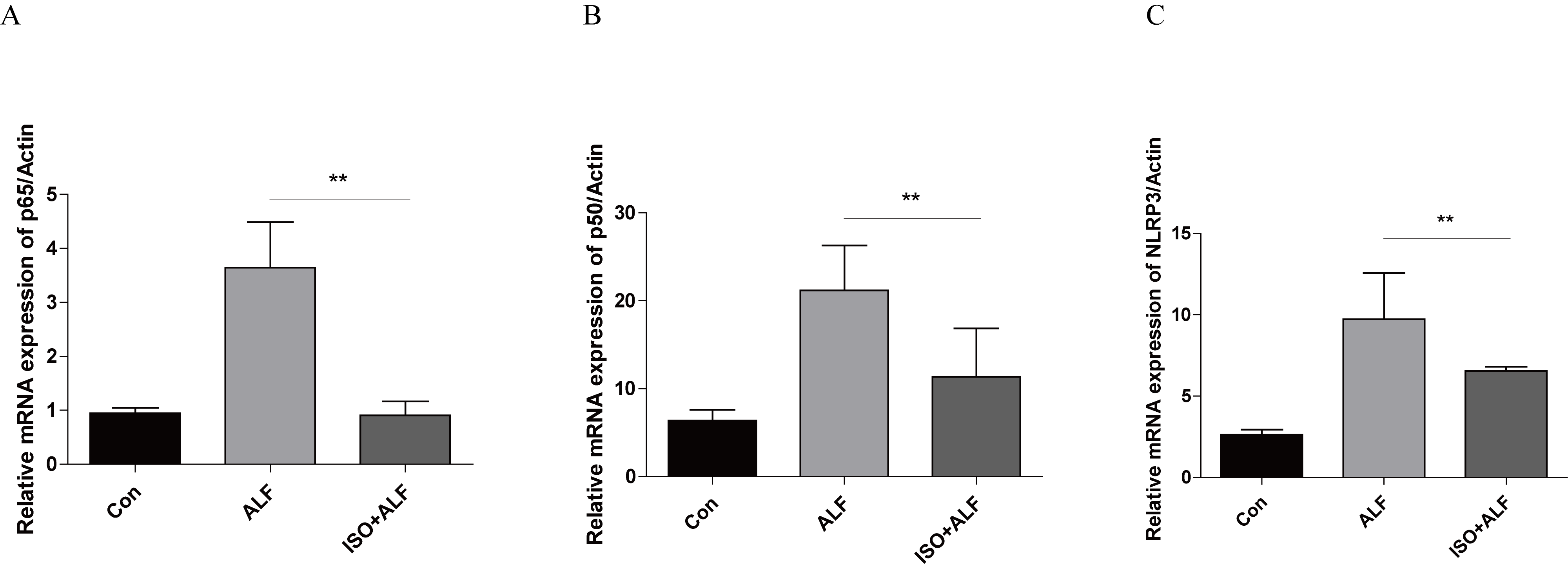

Supplement: Supplementary file 6 — Figure S5. RT-qPCR analysis for the mRNA expression of hub proteins related to MAPK14 and NF-κB pathways in isolated liver non-parenchymal cells. Relative mRNA expression of p65 (A), p50 (B) and NLRP3 (C). Data are presented as mean ± SEM. Statistical significance was performed by Student’s t-test between two groups. **P < 0.01. (PNG 68 KB) [file 10753_2023_1941_MOESM6_ESM.png]

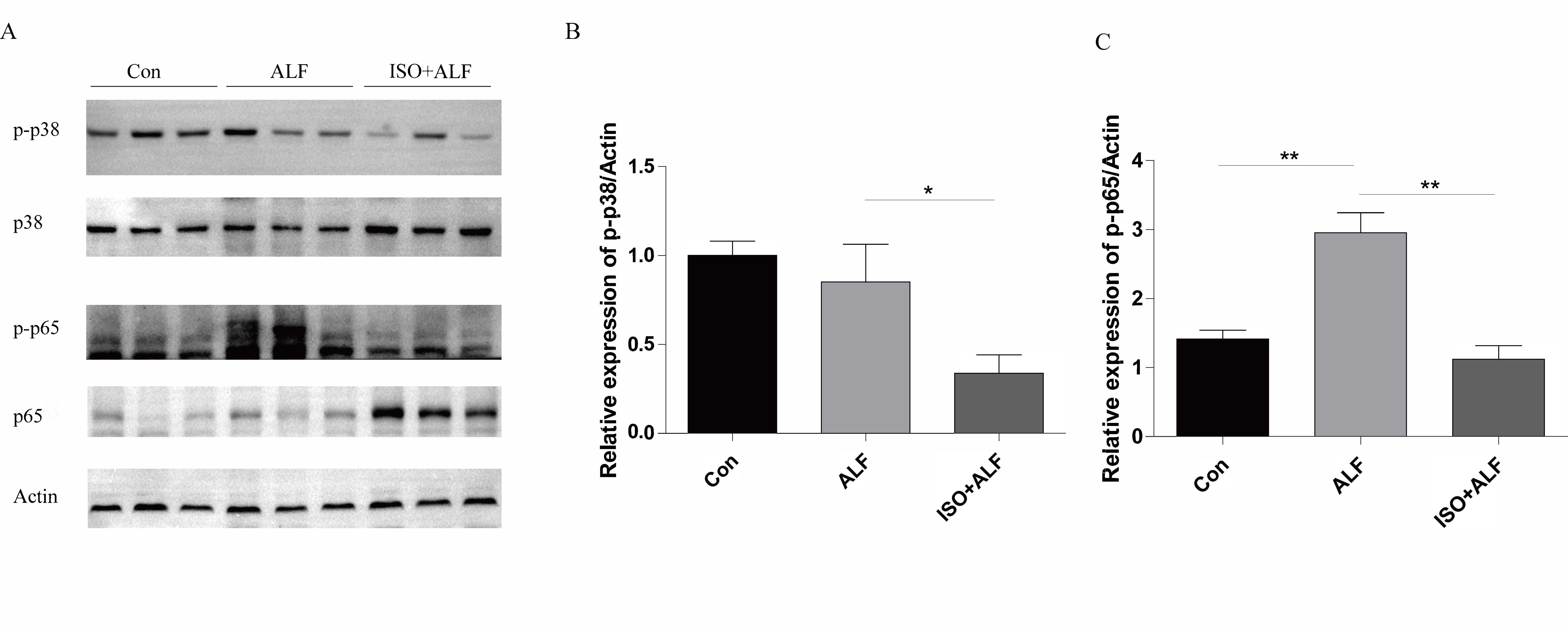

Supplement: Supplementary file 7 — Figure S6. Detection of hepatocyte inflammation in mice liver. (A) Western blot for the protein expressions of inflammation signaling pathways in isolated parenchymal liver cells. The relative expression of phosphorylated p38 (B) and phosphorylated p65 (C) were calculated with bar charts. *P < 0.05; **P < 0.01. (JPG 635 KB) [file 10753_2023_1941_MOESM7_ESM.jpg]
